# HIBAG—HLA genotype imputation with attribute bagging

**DOI:** 10.1038/tpj.2013.18

**Published:** 2013-05-28

**Authors:** X Zheng, J Shen, C Cox, J C Wakefield, M G Ehm, M R Nelson, B S Weir

**Affiliations:** 1 Department of Biostatistics, University of Washington, Seattle, WA, USA; 2 Quantitative Sciences, GlaxoSmithKline, Research Triangle Park, NC, USA; 3 Quantitative Sciences, GlaxoSmithKline, Stevenage, UK

**Keywords:** HLA, MHC, imputation, GWAS, HLA*IMP, BEAGLE

## Abstract

Genotyping of classical human leukocyte antigen (HLA) alleles is an essential tool in the analysis of diseases and adverse drug reactions with associations mapping to the major histocompatibility complex (MHC). However, deriving high-resolution HLA types subsequent to whole-genome single-nucleotide polymorphism (SNP) typing or sequencing is often cost prohibitive for large samples. An alternative approach takes advantage of the extended haplotype structure within the MHC to predict HLA alleles using dense SNP genotypes, such as those available from genome-wide SNP panels. Current methods for HLA imputation are difficult to apply or may require the user to have access to large training data sets with SNP and HLA types. We propose HIBAG, HLA Imputation using attribute BAGging, that makes predictions by averaging HLA-type posterior probabilities over an ensemble of classifiers built on bootstrap samples. We assess the performance of HIBAG using our study data (*n*=2668 subjects of European ancestry) as a training set and HLA data from the British 1958 birth cohort study (*n*≈1000 subjects) as independent validation samples. Prediction accuracies for *HLA*-*A*, *B*, *C*, *DRB1* and *DQB1* range from 92.2% to 98.1% using a set of SNP markers common to the Illumina 1M Duo, OmniQuad, OmniExpress, 660K and 550K platforms. HIBAG performed well compared with the other two leading methods, HLA*IMP and BEAGLE. This method is implemented in a freely available HIBAG R package that includes pre-fit classifiers for European, Asian, Hispanic and African ancestries, providing a readily available imputation approach without the need to have access to large training data sets.

## Introduction

The human leukocyte antigen (HLA) system, located in the major histocompatibility complex (MHC) on chromosome 6p21.3, is highly polymorphic. This region has been shown to be important in human disease, adverse drug reactions and organ transplantation.^1^ HLA genes have a role in the immune system and autoimmunity as they are central to the presentation of antigens for recognition by T cells. As they have to provide defense against a great diversity of environmental microbes, HLA genes must be able to present a wide range of peptides. Evolutionary pressure at these loci has given rise to a great deal of functional diversity. For example, the *HLA-B* locus has 1898 four-digit alleles listed in the April 2012 release of the IMGT-HLA Database^2^ (http://www.ebi.ac.uk/imgt/hla/).

Classical HLA genotyping methodologies have been predominantly developed for tissue typing purposes, with sequence-based typing (SBT) approaches currently considered the gold standard. Although there is widespread availability of vendors offering HLA genotyping services, the complexities involved in performing this to the standard required for diagnostic purposes make using a SBT approach time-consuming and cost-prohibitive for most research studies wishing to look in detail at the involvement of classical HLA genes in disease. Previous studies have suggested that the existence of some HLA alleles can be predicted by a single-nucleotide polymorphism (SNP)-based tagging approach.^3, 4^ However, SNP-based tagging does not offer a definitive solution to HLA genotyping by prediction as many HLA alleles are found on multiple haplotype backgrounds^5^ that differ among populations.

An alternative to tagging is to use more SNP information to impute HLA types. Multiple methods have been developed for this problem, including LDMhc,^5, 6^ as well as applying general genotype imputation methods such as BEAGLE.^7^ To be effective, these methods require access to a large and ethnically diverse training data set with both SNP and HLA alleles genotyped. To impute HLA types from multiple SNP markers, Leslie *et al.*^5^ used an identity-by-descent model based on approximate coalescent models^8^ to develop their LDMhc algorithm, and used a leave-one-out cross-validation scheme for SNP selection. Dilthey *et al.*^6^ subsequently developed integrated software HLA*IMP for imputing classical HLA alleles from SNP genotypes based on LDMhc, with a modified SNP selection function that leads to pronounced increases in call rate. A training set of SNP haplotypes with known HLA alleles are required by LDMhc, as well as a fine genetic map of the region,^6^ whereas most experimental techniques for determining SNPs provide genotypes rather than haplotypes. Inferring haplotypes from genotypes can be done with the statistical method of approximating coalescent models, PHASE,^9^ or newer algorithms like fastPHASE,^10^ MACH^11^ and IMPUTE2.^12^

BEAGLE, an alternative imputation method to the approximate coalescent approach, allows for the prediction of multiallelic loci.^7^ It locally clusters the observed haplotypes at each position, based on similarity of the haplotypes at markers in the local vicinity.^13^ It is a computationally efficient approach with high accuracy for thousands of samples and markers. Recently, it was used for HLA imputation in a genetic association analysis.^14^ That study illustrates how imputation of functional variation can help fine-map association signals in the MHC.

**Figure 1:**
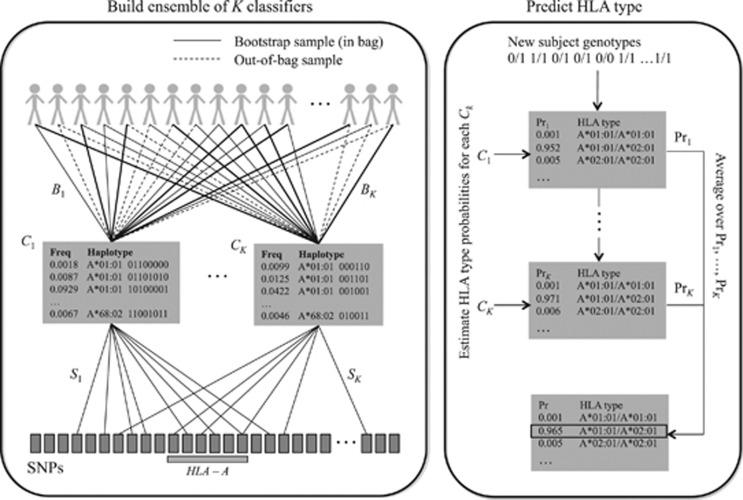
Overview of the HIBAG prediction algorithm. HIBAG is an ensemble classifier consisting of individual classifiers (*C*_*k*_) with human leukocyte antigen (HLA) and single-nucleotide polymorphism (SNP) haplotype probabilities estimated from bootstrapped samples (*B*_*k*_) and SNP subsets (*S*_*k*_). The SNP subsets are determined by a variable selection algorithm with a random component. HLA-type predictions are averaged over the posterior probabilities from all classifiers.

Here we propose a new method for HLA Imputation using attribute BAGging, HIBAG, that is highly accurate, computationally tractable and can be used with published parameter estimates, eliminating the need to access large training samples. It combines the concepts of attribute bagging with haplotype inference from unphased SNPs and HLA types. Attribute bagging is a technique for improving the accuracy and stability of classifier ensembles deduced using bootstrap aggregating and random subsets of variables,^15, 16, 17^ as shown in Figure 1. In this case, individual classifiers are created that utilize a subset of SNPs to predict HLA types and haplotype frequencies estimated from a training data set of SNPs and HLA types. Each of the classifiers employs a variable selection algorithm with a random component to select a subset of the SNPs. HLA-type predictions are determined by maximizing the average posterior probabilities from all classifiers. Compared with LDMhc and BEAGLE, HIBAG has only the minimal assumption of Hardy–Weinberg equilibrium (HWE).

We investigate the overall performance of HIBAG using HLA types and SNP genotypes from HapMap, the British 1958 birth cohort data of the Wellcome Trust Case Control Consortium (WTCCC) and HLARES data from GlaxoSmithKline (GSK) clinical trials. We compare HIBAG with two leading methods, HAP*IMP and BEAGLE v3.3. We provide parameter estimates based on our HLA data and software, implementing our method in the freely available HIBAG R package.

## Materials and methods

The numbers of individuals with available four-digit HLA types and the numbers of observed HLA alleles are summarized in Table 1 for the HapMap, WTCCC and HLARES, respectively. Note that sample sizes vary among HLA loci due to missing data. Descriptions of these data follow.

### HapMap data

The HapMap Phase 2 SNP data set consists of (1) 30 parent–offspring trios of Yoruban ancestry from Ibadan in Nigeria, YRI; (2) 30 CEPH trios of European ancestry from Utah, CEU; (3) 45 unrelated Han Chinese from Beijing, CHB; and (4) 45 unrelated individuals from Tokyo in Japan, JPT. The HapMap SNP genotypes (release #28) were downloaded from http://hapmap.ncbi.nlm.nih.gov/downloads/genotypes/2010-08_phaseII+III/forward/. The data set was created by combining genotyping data from several platforms: Affymetrix, Illumina, Perlegen, and so on. When Mendelian errors were detected in a trio, all genotypes for that SNP in that trio were set to missing. SNP markers were selected within the extended MHC (xMHC)^18^ on chromosome 6 ranging from 025759242 to 033534827 bp. With a missing call rate threshold of 10%, there were 16 241, 17 160 and 16 896 SNP markers in the xMHC for CEU, YRI and CHB+JPT, respectively.

High-resolution classical HLA data for *HLA*-*A*, *B*, *C*, *DRB1*, *DQA1*, *DQB1* and *DPB1* were derived by combining genotypes previously published for these samples^3^ with SBT data generated by Conexio Genomics (Perth, WA, Australia).

### WTCCC data

SNP and HLA genotypes for the British 1958 birth cohort (http://www.b58cgene.sgul.ac.uk/) were downloaded from the European Genotype Archive (http://www.ebi.ac.uk/ega/). Candidate SNP markers from Illumina Human1M-Duo platform^19^ were selected within the xMHC with a 10% threshold of missing SNP genotypes. The final data set included 2922 unrelated individuals and 7601 SNP markers. The HLA data description is available at https://www-gene.cimr.cam.ac.uk/todd/public_data/HLA/HLA.shtml. Five HLA loci, *HLA*-*A*, *B*, *C*, *DRB1* and *DQB1*, were typed to four digits using the Sequence Specific Oligonucleotide (SSO) and Sequence Specific Primer (SSP) methodologies.

### HLARES data

SNP data from the xMHC typed using the Illumina 1M and 1M Duo platforms and HLA data were aggregated from several GlaxoSmithKline clinical trials, including subjects of European (*n*=2668), Asian (*n*=720), Hispanic (*n*=439) and African (*n*=173) ancestries. There were 7976 xMHC SNP markers available with <10% missing data. HLA data for GSK clinical trial samples were generated by Conexio Genomics, HistoGenetics (Ossining, NY, USA) and LabCorp (Burlington, NC, USA) using the SBT, SSO and SSP methodologies for *HLA*-*A*, *B*, *C*, *DRB1*, *DQA1*, *DQB1* and *DPB1*.

### Data for performance assessment

To assess HIBAG performance and build broadly applicable classifiers included in the HIBAG R package, a set of 1564 SNP markers within the xMHC were selected that were available in all the three samples and common to the Illumina 1M Duo, OmniQuad, OmniExpress, 660K and 550K platforms. In the sensitivity analysis, 5316 SNPs within the xMHC genotyped on Illumina 1M Duo platform were used.

The individuals of this study were self-reported as being of European, Asian, Hispanic or African descent. HLA and SNP genotypes for performance assessment (hereafter referred to as ‘STUDY Data’) consist of (1) HLARES data of European ancestry, (2) HLARES data of Asian ancestry and HapMap CHB+JPT, (3) HLARES data of Hispanic ancestry, and (4) African American HLARES data and 60 African parents from HapMap YRI.

## The HIBAG method

We propose the HIBAG algorithm to impute HLA types, using the bagging method developed by Breiman,^15, 20^ with improvements of variable subset suggested by Breiman^16^ and Bryll *et al.*,^17^ applied to a haplotype-based classifier. By randomly sampling sets of individuals from a training data set and randomly selecting SNPs from the available SNPs (as is done in the random forest method), we end up with an ensemble classifier that performs well in predicting HLA types. Here we provide a heuristic description of the process and leave algebraic and algorithmic details in the Appendix. We describe how we develop a set of classifier predictors and then how a user may apply these predictors for a particular individual.

We begin with a set of individuals *T* that have both HLA alleles and SNPs genotyped in the xMHC and we take a series of *K* bootstrap samples (with replacement), *B*_*k*_, of individuals from this set, *k*=1, 2, …, *K*. Each *B*_*k*_ is of size *n*, including some individuals from *T* who appear more than once and some who do not appear at all. Unselected samples form an ‘out-of-bag’ set for the *k*th selection. Breiman^21^ pointed out that about 1/*e*≈37% of *T* are out-of-bag for any *B*_*k*_. We construct a classifier *C*_*k*_ for *B*_*k*_ that estimates HLA types using an optimal subset, *S*_*k*_, of the SNPs. In the following sections, we describe construction of the classifiers *C*_*k*_ and selection of the SNP set *S*_*k*_.

### Individual classifiers

HLA and SNP genotypes available for individuals for each bootstrap sample *B*_*k*_ from *T* are used to form haplotypes and their estimated frequencies (Figure 1) using the EM algorithm assuming HWE^22^ as extended to multiple loci:^23, 24^ multi-locus genotype frequencies are assumed to be the products of haplotype frequencies. As the number of possible resolutions of phase increases exponentially with the number of heterozygous loci, a progressive ligation computational strategy^25^ is used, in which rare haplotypes with frequency <10^−5^ are ignored in order to achieve a computationally tractable algorithm.

The individual classifier *C*_*k*_ is built using the probability of all possible HLA types given the SNP profile observed at *S*_*k*_. The conditional probability follows from the joint probability of an HLA type and the SNP genotypes, and this, in turn, is the sum, over all pairs of haplotypes that are consistent with the observed genotypes, of the products of frequencies of those two haplotypes. For example, HLA heterozygote *A*_1_*A*_2_ and one-locus SNP heterozygote profile *s*_1_*s*_2_ requires summation over two pairs of haplotypes (*A*_1_*s*_1_,*A*_2_*s*_2_) and (*A*_1_*s*_2_,*A*_2_*s*_1_).

### SNP selection

In building each classifier, we select a subset *S*_*k*_ of SNPs for predicting HLA types to reduce overfitting and assure a computationally tractable method. The selection of *S*_*k*_ includes a random and a deterministic component, iteratively sampling a subset *m*_try_ of the *m* total SNPs at random, adding each of the *m*_try_ SNPs to *C*_*k*_ one at a time, and adding the SNP that results in the highest out-of-bag prediction accuracy to *S*_*k*_. This process is repeated, adding one SNP at a time to *S*_*k*_, until no further improvement in prediction of HLA types is achieved by adding additional SNPs. In our study, the size of *S*_*k*_ ranged from 24 to 56 SNPs.

We set *m*_try_ to be much less than *m* (the total number of SNPs) to increase the independence of individual classifiers and reduce the variance of the ensemble by distributing classifiers semi-randomly over all SNPs. If *m*_try_ is too small compared with *m*, the variable selection approach is likely to select less-informative SNP markers. Although this would not necessarily reduce accuracy, it would require larger numbers of classifiers. In general, reducing *m*_try_ reduces both the correlation and the strength of individual classifiers, whereas increasing it increases both. We have found a value of 
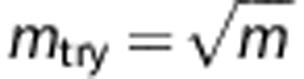
 to perform well, as shown in Supplementary Table S1. This rule is a recommendation of the random forest method (Hastie *et al.*,^26^, Section 15.3).

### Bootstrap aggregation

HIBAG is an ensemble classifer that employs bootstrap aggregation, known as bagging. The ensemble classifier is created from *K* bootstrap samples, each using a different set *S*_*k*_ of SNPs to build a single classifier *C*_*k*_. We have found that *K*=25 is generally sufficient to provide good performance, although we use *K*=100 below in Results to maximize or stabilize prediction accuracies. A comparison of accuracies among different model parameters *m*_try_ and *K* is shown in Supplementary Table S1.

Application of HIBAG to a subject with the observed SNP genotypes estimates the probability of each possible HLA type for all *K* classifiers. The process of aggregating (averaging over) the *K* predictors results in greater precision in the prediction probabilities. In this study, we choose the HLA type with the highest probability averaged over the *K* probabilities as the final predicted genotype for estimating measures of prediction quality. However, in other applications, such as in the analysis of genotype–phenotype relationships, the vector of genotype probabilities may be preferred.

### Implementation

We implemented the algorithm in an R package—HIBAG, which is available at R CRAN (http://cran.r-project.org/web/packages/HIBAG/index.html). To facilitate future use of this method, we have prepared pre-built classifiers based on STUDY Data (described in ‘Data for performance assessment’), which can be used to impute HLA alleles in new SNP data, which are available at http://www.biostat.washington.edu/bsweir/HIBAG/. These classifiers were constructed using the training data sets, as reported in this paper with supporting test results. This enables users to apply the HIBAG method without needing access to a training data set. Alternatively, the software can build new classifiers from training data supplied by the user, and HIBAG is computationally feasible for much large training samples. As the construction of individual classifiers is independent from each other, building an ensemble model in parallel is possible. As an example, it takes about 52 min to build an individual classifier of *HLA*-*A* on the training samples of European ancestry data (*n*=1504) with 273 SNP markers on average. More details are shown in Supplementary Table S2. The computation time while using the published parameters is much less, for example, the algorithm takes at most 41 min for predicting 100 new individuals at *HLA*-*B* locus, as no training is needed.

## Results

We evaluated the performance of HIBAG by building the classifier using a training sample and imputing HLA types in an independent testing sample and compared the imputed genotypes with experimentally determined HLA types. As a further evaluation, we compared the performance of HIBAG with HLA*IMP and BEAGLE.

### Measures of prediction quality

Prediction accuracy was used to assess overall model performance, defined as ‘the number of chromosomes with HLA alleles predicted correctly’ over ‘the total number of chromosomes’. In addition, sensitivity, specificity, positive predictive value and negative predictive value were used to evaluate the predictive performance for each HLA allele. These standard statistical quantities are defined in Supplementary Figure S1. HIBAG produces a posterior probability for each possible HLA type. Placing a minimum threshold on the posterior genotype probability can increase prediction accuracy at the expense of reducing call rates. ‘Call’ and ‘No Call’ were determined by whether the posterior probability is greater or less than a call threshold (CT).

### Accuracy of imputed HLA types on individuals of European ancestry

We compared imputed with experimentally determined HLA types for European ancestries. The HIBAG models were built using the HLARES samples of European ancestry as the training data, and the imputation accuracy was assessed with the independent testing data of the British 1958 birth cohort study. We used the set of 1564 MHC SNPs in common among several Illumina platforms for this analysis. Flanking regions from 50 to 1000 kb were evaluated to identify an appropriate size for predicting HLA alleles, and we conservatively chose to use a 500-kb flanking region, including 1042 SNPs, for our published pre-fit classifiers (Supplementary Figure S2).

The locus-specific calling accuracies were estimated from independent testing data sets (Table 2). In Europeans, without any CT (CT=0) the accuracies range from 92.2% to 98.1% at the five HLA loci. *HLA*-*A* and *DQB1* yielded the highest prediction accuracies, closely followed by *B* and *C*. The lowest accuracy was observed for *DRB1*. We next investigated the influence that setting CTs on posterior probabilities has on calling accuracy and the trade-off this imposes on call rates. The prediction accuracies can be improved by taking the HIBAG posterior genotype probabilities into account with an appropriate CT, and the improvement in accuracy comes at a cost of lower genotype call rates (94.6–99.5%).

In order to compare the performance with the Oxford HLA imputation framework, HLA*IMP, HLARES data of European ancestry were employed as independent validation samples. The HLA*IMP method is implemented in a web-based application with access to a training data set consisting of HapMap 30 CEU trios, the British 1958 birth cohort data of WTCCC and a small number of additional samples from other projects.^6^ Furthermore, using the Illumina 1M option for HLA*IMP, we were able to identify SNPs used for prediction (Supplementary Table S4). To enable a fair comparison, the training data set for HIBAG was limited to the HapMap 30 CEU trios and the British 1958 birth cohort data with the 191 SNPs selected by HLA*IMP for prediction. To illustrate the advantages of utilizing additional SNPs, we provide accuracy results for all the Illumina 1M SNPs using the same training subjects. Results are summarized in Table 3. On the same set of 191 SNPs, HIBAG outperformed HLA*IMP at each locus, especially for *HLA*-*A* (accuracy=96.7% versus 91.0%, respectively). As expected, using more SNP predictors yielded more accurate predictions, although the gains were fairly modest.

### Cross-validation of accuracy for four ancestries

Except for the 1958 birth cohort study, we did not have more data for independent validation; therefore cross-validation was also conducted with respect to each ethnic group: European, Asian, Hispanic and African. For each ethnicity, we divided STUDY Data (defined in ‘Data for performance assessment’) into equal-sized training and validation data sets. The random partitioning strategy produced training and validation data sets with approximately the same numbers of copies of chromosomes with the same HLA alleles. A set of 1564 MHC SNPs in common among several Illumina platforms was used for this analysis. We evaluated flanking regions from 50 to 1000 kb to identify an appropriate size for predicting HLA alleles. In subjects of European ancestry, the average accuracies reach their maximum values by 250 kb (Supplementary Figure S3). We conservatively chose to use a 500-kb flanking region, including 1042 SNPs, for subsequent imputation of all ancestries.

We next investigated the influence that setting CTs on posterior probabilities has on calling accuracy and the trade-off this imposes on call rates. Using 500 kb of flanking markers around each HLA locus, 
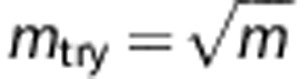
 as the number of markers randomly sampled in building each classifier and *K*=100 bootstrap samples, we built the HIBAG models with European, Asian, Hispanic and African ancestry training data sets, respectively. As shown in Table 4, in Europeans where we have the largest sample size, without any CT (CT=0) the accuracies range from 92.1% to 98.8%. *HLA*-*C* and *DQB1* yielded the highest prediction accuracies, closely followed by *A*, *DQA1* and *B*. The lowest accuracies were observed for *DPB1* and *DRB1*. Among non-Europeans, per locus accuracies were uniformly lower than in Europeans and varied substantially from locus to locus. On average, the prediction accuracy was the lowest in subjects of African ancestry. These patterns are due to the differences in training sample size and several aspects of allelic heterogeneity, including the number of alleles, their frequency distribution and the degree of haplotypic mosaicism within four-digit alleles.^5^ The results using all Illumina 1M MHC markers were not noticeably better than the intersection across several commonly used Illumina genome-wide panels (Supplementary Table S3). We therefore focused on the intersection as a more broadly applicable panel.

**Figure 2:**
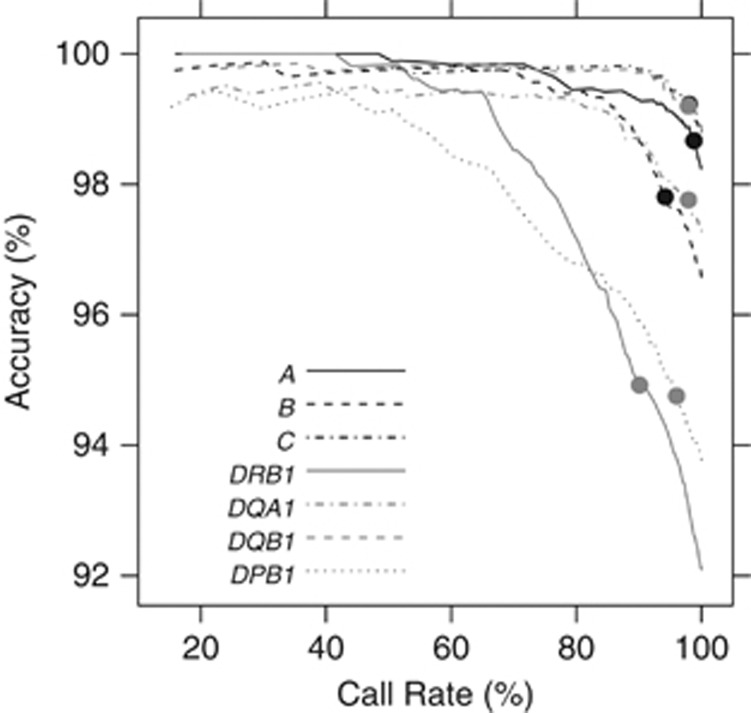
The relationship between accuracy and call rate when HLARES data for individuals of European ancestry are divided into training and validation sets with equal sizes. On the curve for each HLA (human leukocyte antigen) locus, the 0.5 call threshold is indicated by •.

The prediction accuracy can be improved by taking the HIBAG posterior genotype probabilities into account, as those with higher probabilities have a higher likelihood of being a correct call. An empirical relationship between posterior probability and overall accuracy is shown in Supplementary Figure S4. The improvement in accuracy comes at a cost of lower genotype call rates, as illustrated in Figure 2 and Supplementary Figure S5. All seven loci can achieve >99% calling accuracy with sufficiently stringent choices of posterior probabilities; however, this would lead to call rates <60% in the case of *DRB1* and *DPB1*. The best choice of CT for each locus will vary based on study criteria. We have selected a threshold of 0.5 as a value that has modest effects on both call rate and accuracy. At this threshold, the accuracy range in Europeans increases from 94.8% to 99.2%, with call rates between 90.1% and 98.8%. Among the non-Europeans, in some instances this threshold led to dramatic improvements in accuracies with corresponding decreases in call rates. For example, the accuracy of *HLA*-*B* types in subjects of African ancestry improved from 76.8% to 96.7%, but with a call rate of only 21.1%. This highlights the importance of careful CT selection.

The performance summaries by HLA locus presented above are an average of the accuracies of each of the alleles observed in the testing data set, weighted by their corresponding frequencies. Details of the predictive characteristics of each HLA allele using a CT of 0.5 are summarized in Supplementary Tables S5–8. Some alleles have very high accuracies, whereas others are much lower. Alleles with low accuracy tend to have lower frequencies, as illustrated in Supplementary Figure S6. Our study confirms that having 10 copies of an allele in the database is generally sufficient to provide high sensitivity (>90% except for *HLA*-*B* and *DRB1*).^5^ We found that in most instances where alleles are miscalled, there is one particular allele that is substituted for the correct one (Supplementary Table S5). For example, *HLA*–DRB1*01:01 has an 8% allele frequency in Europeans and is miscalled just over 5.6% of the time. In every instance that DRB1*01:01 is miscalled, it is called as DRB1*01:02. This miscall is reasonable as DRB1*01:01 and DRB1*01:02 both belong to the same serological antigen carried by an allotype DRB1*01.

BEAGLE is commonly used for genotype imputation and is unique among commonly used methods by accommodating multi-allelic variants.^7^ It has been used to impute HLA types.^14^ We therefore compared the performance of HIBAG with BEAGLE v3.3 (Table 4 and Supplementary Table S3). The default settings for BEAGLE were used, except that we increased the number of iterations from 10 to 50, which improves prediction accuracies. Note that the manner of applying BEAGLE in this study is different from that in Raychaudhuri *et al.*^14^ We applied BEAGLE to impute HLA alleles gene by gene with flanking SNPs, as the efficiency of BEAGLE could be improved by restricting the number of SNPs that are possibly included in the model.

As BEAGLE does not provide posterior probabilities for predicted HLA types, we compared BEAGLE’s imputed HLA types with HIBAG’s HLA types assuming no CT. As shown in Table 4, the prediction accuracies of HIBAG and BEAGLE are similar. For samples of European ancestry, BEAGLE yields higher prediction accuracies than HIBAG at *HLA*–*DRB1* and *DPB1* (92.9% versus 92.1% and 94.7% versus 93.8%, respectively). However, HIBAG performed better at all other loci. For the non-European ancestries, the accuracies of BEAGLE and HIBAG are similar. A clear advantage of HIBAG over BEAGLE in the context of imputing HLA types is that HIBAG can be run efficiently using published classifiers, whereas BEAGLE requires a training data set.

## Discussion

We propose HIBAG, an ensemble classifier, for the imputation of HLA types from dense SNP data. The HIBAG classifier consists of individual classifiers and makes a prediction by averaging HLA-type posterior probabilities over the collection. Our comparisons indicate that HIBAG performs marginally better than HLA*IMP developed by Dilthey *et al.*^6^ and is comparable with BEAGLE. HIBAG prediction accuracies for individuals of European ancestry range from 94.8% to 99.2% when using a CT of 0.5 with a subset of SNPs common to several popular Illumina platforms.

Studies that identify significant associations within the MHC may be limited by the high cost of typing required to investigate the contributions of underlying HLA alleles. Our SNP-based method provides an efficient way of imputing HLA types using genome-wide genotype data. A previous study has indicated that MHC-class-I-mediated events, principally involving *HLA*-B*39, contribute to the etiology of type 1 diabetes.^27^ HLA alleles are associated with some of the strongest adverse drug reactions, for example, B*57:01 with Abacavir, which is used to treat HIV and AIDS,^28^ and B*58:01 with Allopurinol used primarily to treat hyperuricemia.^29^ Our results show that the predictions of B*57:01 and B*58:01 have 100% sensitivities and specificities with call rates >95% for Europeans.

HIBAG produces the posterior probability of each HLA type. A direct application is to use the best-guess genotypes and CT in downstream association analysis, such as an additive logistic regression model.^14^ As shown in Supplementary Figure S4, individuals with higher posterior probabilities have a higher likelihood of being a correct call, and a CT of 0.5 approximately corresponds to a prediction accuracy of 80%. An alternative could be to model the uncertainty of prediction via posterior probabilities.

Our method and parameter estimates are freely available in the HIBAG R package. A typical parameter file for imputing HLA types contains only haplotype frequencies at different SNP subsets rather than individual training genotypes. Further, unlike the web-implemented HLA*IMP, HIBAG does not require the uploading of genotype information to a website, which could raise concerns over data privacy, or having access to large training HLA data sets. To facilitate future use of this method, we have prepared pre-fit classifiers based on STUDY Data (defined in ‘Data for performance assessment’), which can be used to impute HLA types in new SNP data. The SNP markers selected in the pre-fit classifiers are common to the Illumina 1M Duo, OmniQuad, OmniExpress, 660K and 550K platforms within a 500-kb flanking region of each HLA gene.

In our published pre-fit classifiers, we selected 1042 SNPs in total. However, it is possible to find a smaller set of SNPs without sacrificing accuracy using our method. HLA*IMP has developed a selection approach to identify a small set of most informative SNPs for predicting HLA types. With respect to HIBAG, variable selection is implicitly incorporated during the construction of each individual classifier, and more important SNP markers tend to be used more frequently in the ensemble. This use of selection frequency for SNPs provides information to identify a small set of SNPs. The use frequencies of SNPs in the published pre-fit classifiers are shown in Supplementary Figure S8. SNPs with low importance do not tend to contribute to accuracy. For example, a threshold of 25 classifiers was used to filter out less important SNPs for European ancestry, and the total number of SNP predictors for *HLA*-*A*, *B*,..., *DPB1* changes from 1042 to 779 without reducing accuracy (data not shown).

**Figure 3:**
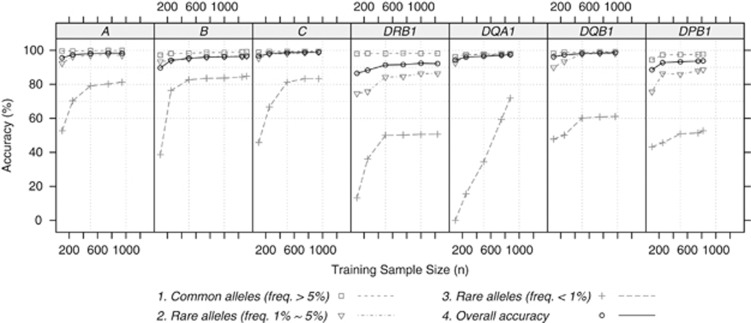
The relationship between training sample size and accuracy. HLARES data of European ancestry were divided into training and validation sets with equal sizes, and random subsets of training samples (*n*=100, 250, 500, 750, 1000 and max) were used to build a HIBAG model, which was applied to the same validation samples. No call threshold was applied.

When HLARES data of European ancestry were investigated, the overall accuracies increase with the training sample size but are only slightly improved after 500 training samples, as shown in Figure 3. Rare alleles with frequency <1% have significantly lower prediction accuracies than the common alleles. The size of sample sets required to accurately type rare alleles using an imputation methodology is impractical. Although we observed 144 unique *HLA*-*B* alleles in our total study population (*n*=5515), typing of >28 000 individuals for *HLA*-*B* by the Nation Marrow Donor Program^30^ identified only 184 unique *HLA*-*B* alleles, still representing <10% of the 1898 four-digit *HLA*-*B* alleles currently identified by IMGT.

The accuracies of common alleles for *HLA*-*A*, *B*, *C* and *DQB1* are >99%, whereas that of *DPB1* is the lowest (∼97%). Possible reasons for imperfect predictions on the alleles of >1% frequency are data quality of genotypes, the ambiguity of HLA alleles due to typing approach, missing SNPs and loss of distinguishable SNP patterns. Leslie *et al.*^5^ did observe chromosomes that have nearly identical SNP patterns, yet carry different HLA alleles.^5^ Denser SNP markers, especially those SNPs in an HLA gene, may increase overall accuracies.

A simulation study indicates that the HIBAG method is robust to missing SNP markers with a fraction up to 50%, as shown in Supplementary Figure S7. The missing SNP fraction of the original validation set is very small (<0.1%). For each simulation run, we randomly remove a fraction of the SNP predictors used in the ensemble classifier (for example, 10, 20%) for the validation set where every validation sample has the same missing SNPs and repeat this procedure 100 times. The box plots of accuracies (CT=0 and 0.5) and call rate are shown. The missing SNPs do not significantly reduce the accuracies for missing fractions <80%, but it does decrease the call rates.

Whether the HIBAG algorithm is sensitive to deviations from HWE was assessed with multi-ethnic samples where HWE does not hold. For each ethnicity, STUDY data were divided into training and validation sets with equal sizes as described in the previous section. A multi-ethnic HIBAG model was built using all training samples from multiple ethnicities, and then the accuracies were calculated for each validation set. As shown in Supplementary Table S9, the prediction accuracies of multi-ethnic models were similar to those of ethnic-specific models without significant decrease; there was even some improvement on accuracies. Furthermore, our algorithm imputes new study subjects one by one; thereby the imputed HLA type of an individual is not affected by the other new subjects. These results indicate that our method is robust to departures from HWE.

HLA*IMP relies on high-quality haplotypes in the training data,^5^ which contain the HLA locus of interest and SNP predictors. However, most experimental techniques for determining SNPs do not provide haplotype information, and the quality of computational phasing of unrelated individuals may not be satisfactory. On the other hand, BEAGLE assumes variable-length Markov chains besides HWE to represent linkage disequilibrium,^31^ which is a bias–variance tradeoff in a possibly very high-dimensional problem.^32^ As linkage disequilibrium in the xMHC typically follows a complex pattern, HIBAG does not make any assumption except HWE and is possibly more suitable to the complex MHC region than methods with additional assumptions.

It is important to realize the potential limitations, and our findings should be interpreted with caution. The numbers of HLA alleles documented in the IMGT-HLA database^2^ are much larger than the numbers investigated in our study. For example, the numbers of four-digit HLA alleles from IMGT are 1365, 1898 and 1006 at the *HLA*-*A*, *B* and *C* loci, respectively, and new alleles are routinely being discovered, but we have only 85, 144 and 49 alleles, respectively, in our training samples. The prediction accuracies reported here are computed from restricted validation samples whose HLA alleles are present in the training set. Quite large training sets might be required to successfully predict most of HLA alleles in the IMGT-HLA data set, as 10 copies of an allele in the training database are generally thought to be required to provide high sensitivity.^5^

In summary, we propose a new method for HLA type imputation with performance similar to existing methods, including HLA*IMP and BEAGLE, with several differentiating factors. The HIBAG and BEAGLE utilize all the available SNPs in the region, which results in increased accuracy for these methods versus HLA*IMP. The freely available HIBAG method and accompanying parameter estimates (published in this paper) enable the method to be applied without the need to upload data to an external website (that is, HLA*IMP) or to have access to a training data set (BEAGLE).

## Figures and Tables

**Table 1:** The numbers of individuals with four-digit HLA types and the observed number of HLA alleles for each locus

	*HLA type*						
	*A*	*B*	*C*	*DRB1*	*DQA1*	*DQB1*	*DPB1*
*Individuals genotyped*							
* HapMap*							
* *CEU	90	68	90	90	90	90	90
* *YRI	90	88	88	89	90	88	12
* *CHB+JPT	89	89	89	88	89	87	58
							
* WTCCC*							
* *European	884	1532	840	1129	0	1004	0
							
* HLARES*							
* *European	1857	2572	1866	2436	1740	1924	1624
* *Asian	517	624	522	608	495	525	469
* *Hispanic	298	430	300	420	269	312	263
* *African	80	112	80	102	74	78	69
							
*Unique HLA alleles*							
* *European	48	88	37	55	17	21	26
* *Asian	43	72	34	49	17	19	29
* *Hispanic	41	85	31	44	14	17	26
* *African	36	45	24	30	13	17	23
* *Total	85	144	49	80	19	27	49

Abbreviations: HLA, human leukocyte antigen; WTCCC, Wellcome Trust Case Control Consortium.

**Table 2:** Summary of the four-digit prediction accuracies (call rates) for HLARES of European ancestry, using four-digit HLA data from the British 1958 birth cohort study as independent validation samples

	*HLA type*				
	*A*	*B*	*C*	*DRB1*	*DQB1*
No. of SNPs^a^	273	341	356	327	356
No. of training samples	1857	2572	1866	2436	1924
No. of validation samples	884	1532	840	1129	1004
					
*HLARES training data of European ancestry, the published pre-fit classifiers:*					
CT=0	98.1 (100)	96.9 (100)	96.5 (100)	92.2 (100)	97.8 (100)
CT=0.5	98.2 (99.4)	97.4 (97.3)	96.6 (99.5)	94.0 (94.6)	98.0 (99.0)

Abbreviations: CT, call threshold; HLA, human leukocyte antigen; SNP, single-nucleotide polymorphism.

HIBAG CT of 0 and 0.5 were used.

a SNP markers common to the Illumina 1M Duo, OmniQuad, OmniExpress, 660K and 550K platforms within a flanking region of 500 kb were used.

**Table 3:** The comparison of four-digit accuracies for HIBAG and HLA*IMP on HLARES data of European ancestry with no call threshold

	*HLA type*				
*Method*	*A*	*B*	*C*	*DRB1*	*DQB1*
No.of validation samples	1787	2471	1830	2383	1917
					
Using 191 markers on Illumina 1M platform as selected by HLA*IMP a					
No.of SNPs	50	39	27	50	34
HLA*IMP (%)	91.0	94.4	98.4	87.9	96.2
HIBAG b (%)	96.7	94.8	98.7	90.0	98.6
					
*Using all the xMHC markers on Illumina 1M platform* c					
No. of SNPs	489	562	554	474	447
HIBAG^b^ (%)	97.7	95.1	98.7	91.8	98.4

Abbreviations: HLA, human leukocyte antigen; MHC, major histocompatibility complex; SNP, single-nucleotide polymorphism.

a The full SNP list is shown in Supplementary Table S4.

b The training samples are HapMap 30 CEU trios plus WTCCC samples.

c The SNP markers within a flanking region of 250 kb are used.

**Table 4:** Summary of the four-digit prediction accuracies (call rates) stratified by ancestries and HLA loci

	*HLA type*						
	*A*	*B*	*C*	*DRB1*	*DQA1*	*DQB1*	*DPB1*
*European ancestry*							
No. of SNPs^a^	273	341	356	327	349	356	279
							
*HIBAG*							
CT=0.0	98.2 (100)	96.6 (100)	98.8 (100)	92.1 (100)	97.3 (100)	98.8 (100)	93.8 (100)
CT=0.5	98.7 (98.8)	97.8 (94.2)	99.2 (98.0)	94.9 (90.1)	97.8 (97.9)	99.2 (97.9)	94.8 (96.0)
							

*BEAGLE* b	98.1 (100)	95.5 (100)	97.7 (100)	92.9 (100)	96.4 (100)	97.9 (100)	94.7 (100)
							
*Asian ancestry*							
No. of SNPs^a^	259	334	346	319	341	348	272
							
*HIBAG*							
CT=0.0	92.1 (100)	87.5 (100)	96.6 (100)	88.7 (100)	86.8 (100)	96.0 (100)	89.8 (100)
CT=0.5	93.8 (91.7)	94.7 (71.0)	97.8 (93.9)	95.8 (71.5)	90.0 (80.8)	98.1 (96.3)	95.3 (82.8)
							

*BEAGLE* b	93.8 (100)	83.7 (100)	94.5 (100)	87.7 (100)	86.7 (100)	97.3 (100)	91.2 (100)
							
*Hispanic ancestry*							
No. of SNPs^a^	274	341	356	326	348	355	278
							
*HIBAG*							
CT=0.0	93.4 (100)	75.0 (100)	96.2 (100)	82.0 (100)	93.8 (100)	95.7 (100)	93.1 (100)
CT=0.5	96.0 (82.5)	93.8 (37.5)	98.4 (87.4)	93.5 (50.8)	95.8 (90.8)	98.9 (90.0)	97.5 (81.5)
							

*BEAGLE* b	89.1 (100)	75.0 (100)	92.3 (100)	78.7 (100)	94.6 (100)	96.3 (100)	91.9 (100)
							
*African ancestry*							
No. of SNPs^a^	266	335	349	325	343	351	269
							
*HIBAG*							
CT=0.0	92.4 (100)	76.8 (100)	88.5 (100)	77.1 (100)	80.0 (100)	79.4 (100)	74.2 (100)
CT=0.5	100 (74.6)	96.7 (21.1)	96.5 (66.2)	100 (22.2)	97.2 (27.7)	97.7 (34.9)	75.0 (12.9)
							

*BEAGLE* b	93.2 (100)	71.1 (100)	86.9 (100)	81.2 (100)	79.2 (100)	76.2 (100)	79.0 (100)

Abbreviations: HLA, human leukocyte antigen; SNP, single-nucleotide polymorphism.

STUDY data were divided into training and validation sets with equal sizes. HIBAG call thresholds (CTs) of 0 and 0.5 were used.

a SNP markers common to the Illumina 1M Duo, OmniQuad, OmniExpress, 660K and 550K platforms within a flanking region of 500 kb are used.

b No call threshold.

## Box 1 The attribute bagging algorithm

1. For *k*=1 to *K*:
  - Draw a bootstrap sample *B*_*k*_ of size *n* (no. of training samples) with replacement from the training data, and *B*_*k*_ contains all candidate SNPs.
  - Build an individual classifier *C*_*k*_ on *B*_*k*_ by variable selection with a random component (in Box 2), where *C*_*k*_ uses only a small set of SNPs *S*_*k*_.
2. Output the ensemble of individual classifiers .
3. To predict the HLA type of a new individual with SNP profile , we average the posterior probabilities  among all *C*_*k*_. The prediction is

## Box 2 The algorithm of variable selection with a random component for an individual classifier *C*_*k*_

1. The set of loci for estimating haplotype frequencies is initially set to .
2. Build an individual classifier *C*_*k*_ on the bootstrapped data *B*_*k*_, by recursively repeating the following steps from (i) to (iii) until it is not possible to reduce the losses^1^:
  Select *m*_try_ SNP markers at random without replacement from the *m* total candidate SNPs (*m*_try_< *m*) except the marker(s) in *S*_*k*_.
  Select the best SNP marker j* based on the criteria of losses^1^ among *m*_try_.
  Add the selected SNP marker to the set .
3. Output *C*_*k*_ and *S*_*k*_.

^1^: reduce both the 0–1 loss and log likelihood loss of *C*_*k*_ (see the section ‘Details of the loss criteria’).

## Appendix

### Individual classifier

Let *y* denote an HLA allele, *x* a SNP allele and *g* a SNP genotype. An HLA type at a specified locus is denoted as an unordered pair of alleles . Let *n* be the total number of samples, *m* be the total number of candidate SNPs and the index *j* (1⩽ *j* ⩽ *m*) the *j*th SNP marker. An individual classifier *C*_*k*_ (*k*=1, 2,…, *K*) is built from the posterior probabilities of HLA types given by the SNP genotypes at a set of loci *S*_*k*_:

where *K* is the total number of classifiers.

The joint probability over SNP genotypes for an HLA type  can be estimated from training genotypes via haplotype frequencies. As unexpected haplotypes sometimes are observed in the genotypes of new individuals due to genotyping error, SNP mutation or rare haplotypes with <10^−5^ frequency, an error rate per locus (10^−5^) is used. Under the HWE assumption, the joint probability is the summation of frequencies of two associated haplotypes:

where the set Ω represents all haplotype pairs of  and  whose genotypes are consistent with the observed ones , and  denotes the frequency of a haplotype .

The haplotype frequencies  are estimated from the training data *T* and lead to estimated conditional probabilities of genotypes

in equation (1). For a new individual with SNP profile , the prediction of *T*_*k*_ is

[Boxed-text box1]

[Boxed-text box2]

### Attribute bagging algorithm

The attribute bagging algorithm is shown in Boxes 1 and 2. *K* is the total number of individual classifiers, and *m*_try_ is the number of variables randomly sampled as candidates for selection ( by default). For each step of adding a new predictor, *m*_try_ variables are re-drawn randomly from the candidate SNPs.

We find in our experiments that *K*=25 is sufficient to give a highly accurate and stable ensemble classifier, and this number of bootstrap replicates was also used by Breiman.^15^ The prediction accuracy is not sensitive to *m*_try_ as all of SNP markers are determined whether or not they are included in the SNP set for each construction of individual classifier. The motivation for selecting *m*_try_<*m* is to increase the independence of individual classifiers and reduce the variance of the ensemble by distributing classifiers randomly over all SNPs. However, if *m*_try_ is too small compared with *m* (for example, *m*_try_=1), the variable selection approach is likely to select non- or less-informative SNP markers. Although this would not necessarily reduce accuracy, it would require larger numbers of classifiers. In general, reducing *m*_try_ reduces both the correlation and the strength of individual classifiers, and increasing it increases both, therefore the optimal range of *m*_try_ could be usually quite wide. In our experiments, the parameter setting  is appropriate for both a small set as well as hundreds of SNP predictors after taking the computational burden into account. In this study, we used the parameter settings *K*=100,  for all the tables and figures.

### Details of the loss criteria:

Each bootstrap sample *B*_*k*_ leaves out 1/*e*≈37% of the training samples, and these left-out samples can be used to form accurate estimates (called out-of-bag estimation).^21^ The 0–1 loss of *C*_*k*_ is calculated from the out-of-bag samples to avoid over-fitting of that individual classifier. We minimize the 0–1 loss first, and if the 0–1 losses equal each other then choose the SNP marker with the lowest log likelihood loss. We add SNP markers until it is not possible to further reduce both the losses. Unlike the traditional variable selection with a penalty for the number of parameters, for example, the Akaike criterion, our approach adds as many SNP predictors as possible to avoid variable searching stopping too early. Although more variables in an individual classifier result in greater computational complexity and larger variance of estimates, bagging and use of different variable subsets help to improve the stability of ensemble classifier.^17, 15^ We therefore control model over-fitting twice, first at the level of the individual classifier and then at the level of aggregation in the ensemble.

The 0–1 loss of *C*_*k*_ is calculated from the out-of-bag samples to avoid over-fitting of that individual classifier,

and the log likelihood loss of *C*_*k*_ is computed to assess fitting the model of haplotype frequencies with the assumption of HWE using the in-bag samples *B*_*k*_,

where the subscript *i* indicates the *i*th individual in *B*_*k*_.
