# Supplementary material for: HIBAG—HLA genotype imputation with attribute bagging
Source: Pharmacogenomics J. 2013 May 28;14(2):192–200. doi: 10.1038/tpj.2013.18 (PMC3772955; doi:10.1038/tpj.2013.18)
Supplement: Supplementary Information [file tpj201318x1.pdf]

Supplementary Materials – Figures

		True HLA allele		
		H	Not *H*	
Prediction	*H*	True Positive	False Positive	**Positive Predictive Value** = # of “true positive” / # of “prediction is *H*”
	Not *H*	False Negative	True Negative	**Negative Predictive Value** = # of “true negative” / # of “prediction is not *H*”
		**Sensitivity** = # of “true positive” / # of “true allele is *H*”	**Specificity** = # of “true negative” / # of “true allele is not *H*”	**Allele Accuracy** = # of “true pos” + “true neg” / grand sum

Figure S1: The standard statistical quantities of prediction quality for a specific HLA allele H: sensitivity, specificity, positive predictive value, negative predictive value and allele accuracy.

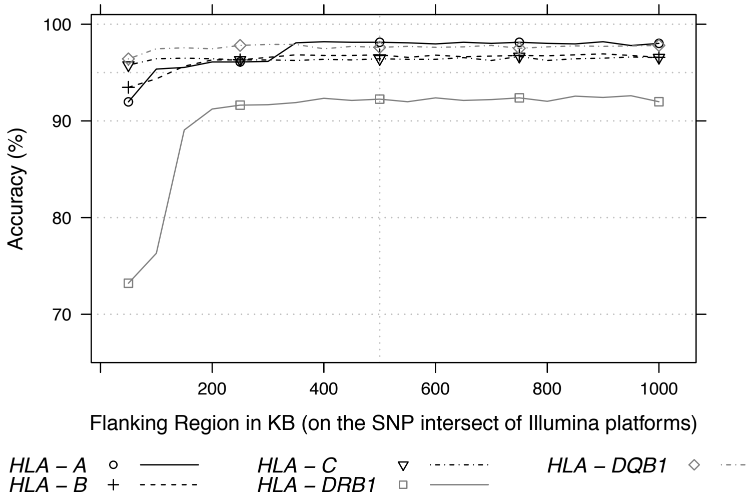

Figure S2: The relationships between the four-digit accuracies (no call threshold) and size of flanking region from 50kb to 1000kb on each side, stratified by HLA loci. The HIBAG models were built using the HLARES samples of European ancestry as the training data, and the imputation accuracies were assessed with the independent testing data of the British 1958 birth cohort study. SNP markers were genotyped on the intersect of Illumina platforms. A 500kb flanking region is an appropriate region for predicting HLA alleles.

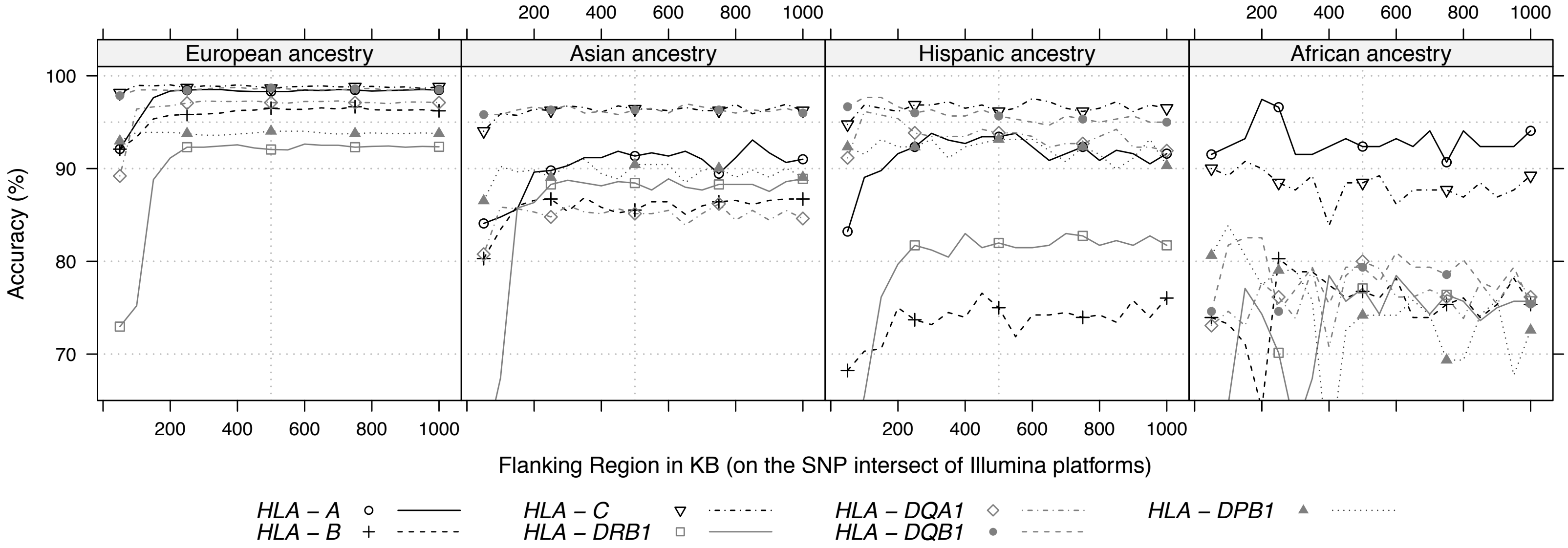

Figure S3: The relationships between the four-digit accuracies (no call threshold) and size of flanking region from 50kb to 1000kb on each side, stratified by HLA loci and ethnicities. STUDY Data were divided into training and validation sets with equal sizes for each ancestry and each HLA gene. SNP markers were genotyped on the intersect of Illumina platforms. A 500kb flanking region is an appropriate region for predicting HLA alleles.

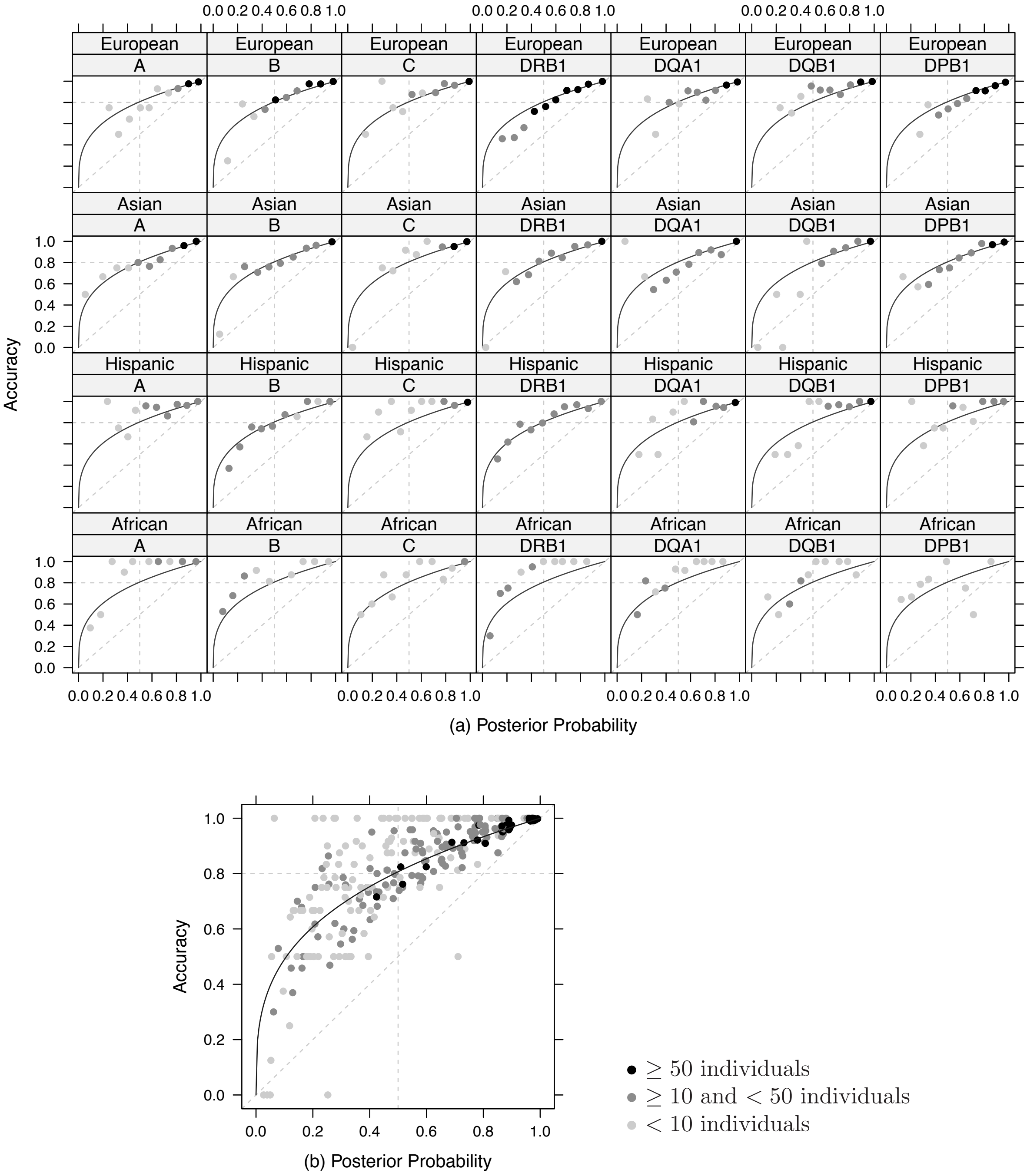

Figure S4: The relationship between posterior probability and overall accuracy. STUDY Data for each ancestry are divided into training and validation sets with equal sizes, and the accuracies are calculated from ten bins of posterior probabilities: (a) stratified by HLA loci and ancestries; (b) over all HLA loci and ancestries, the curve is fitted by a function $y = x^r$ with a parameter $r = 0.31$, and 0.5 posterior probability approximately corresponds to the prediction accuracy 80%.

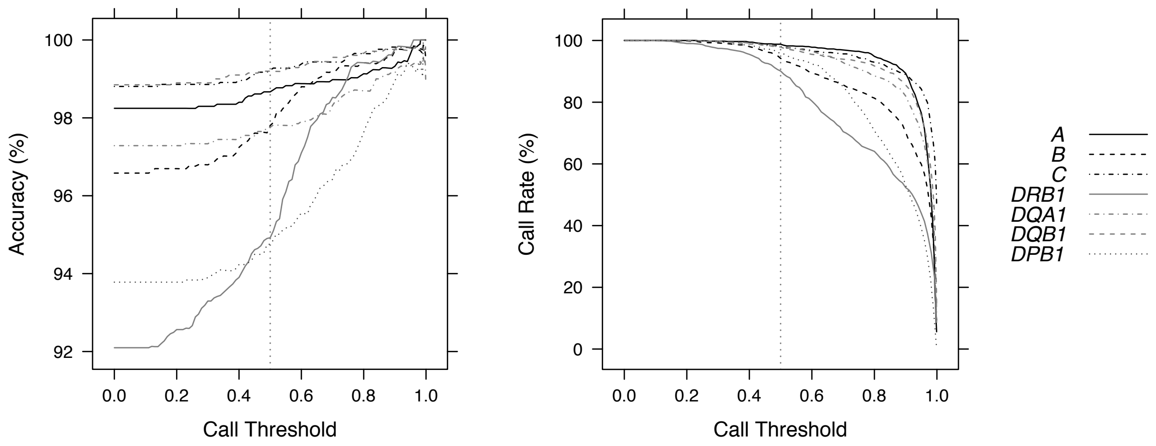

Figure S5: The relationships among call threshold, accuracy and call rate when HLARES data for individuals of European ancestry are divided into training and validation sets with equal sizes.

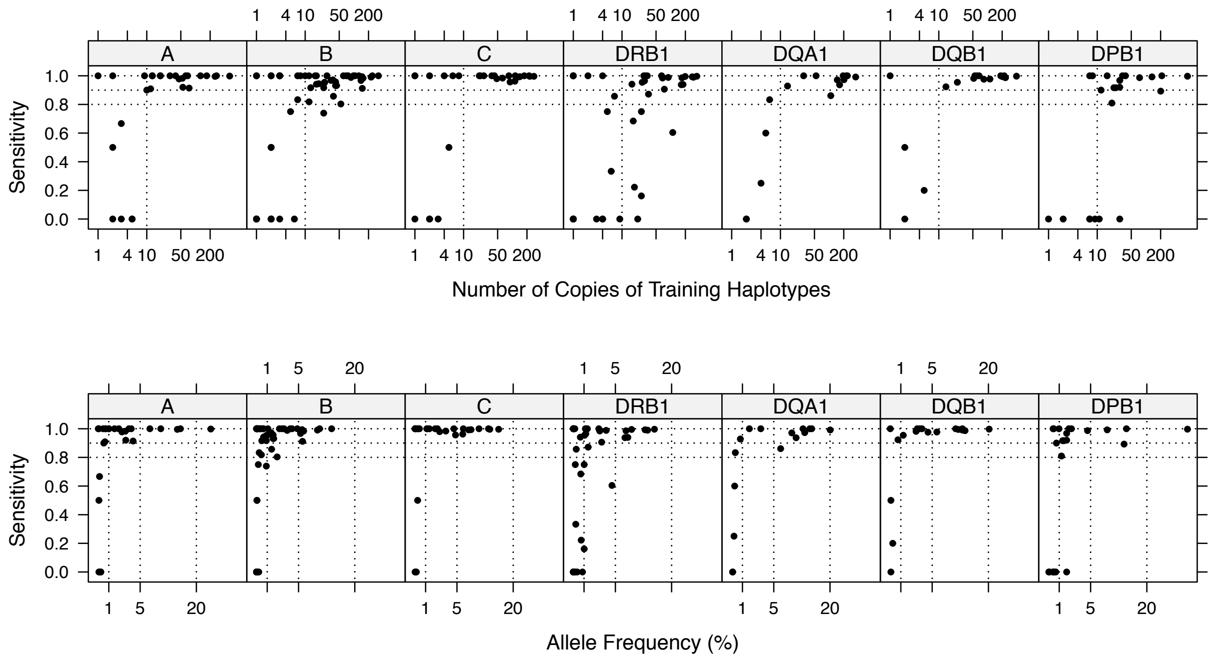

Figure S6: The relationship between four-digit sensitivities (no call threshold) and the number of copies of training haplotypes for each HLA allele when HLARES data for individuals of European ancestry were divided into training and validation sets with equal sizes. SNP markers on the intersect of Illumina platforms were used. For *A, C, DQA1, DQB1* and *DPB1*, 10 copies of training haplotypes seem sufficient to attain 90% sensitivity, but *B* and *DRB1* require many more training haplotypes.

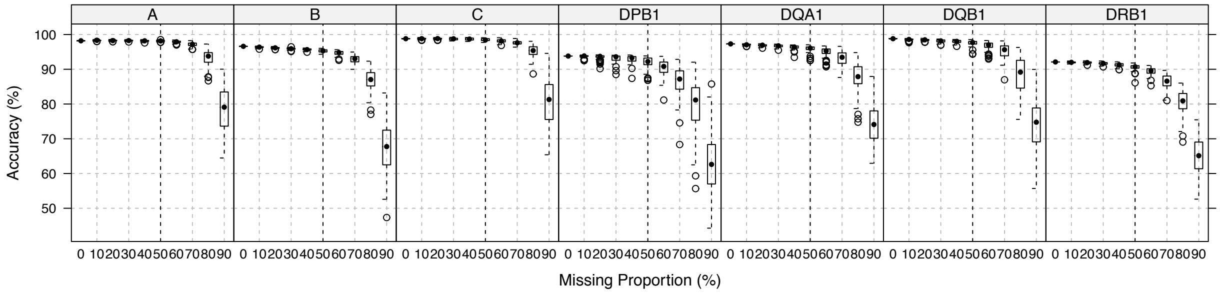

(a) no call threshold: accuracy vs. missing proportion.

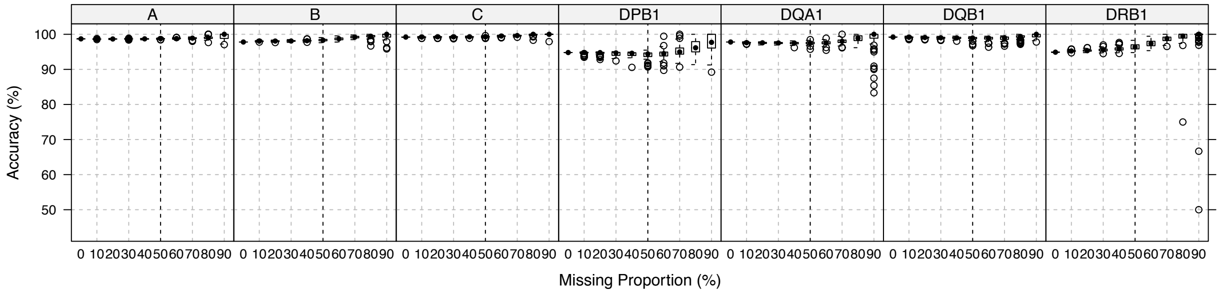

(b) 0.5 call threshold: accuracy vs. missing proportion.

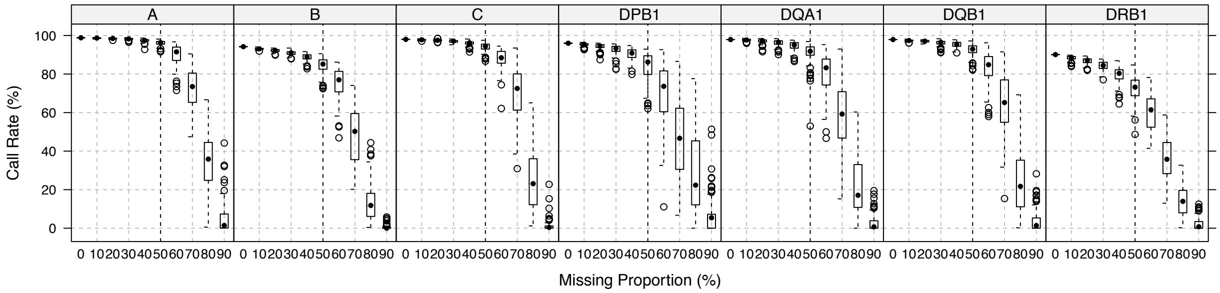

(c) 0.5 call threshold: call rate vs. missing proportion.

Figure S7: Box plots of accuracy and call rate with missing SNPs. HLARES data of European ancestry were divided into training and validation sets with equal sizes. The HIBAG models were built using the training parts. For each run of simulation, a fraction of the SNP predictors used in the ensemble classifier (e.g, 10%, 20%) was removed randomly for the validation set, where every validation sample has the same missing SNPs, and repeat it 100 times. The missing SNPs do not significantly reduce the accuracies for missing fraction $< 80\%$, but it does decrease the call rates.

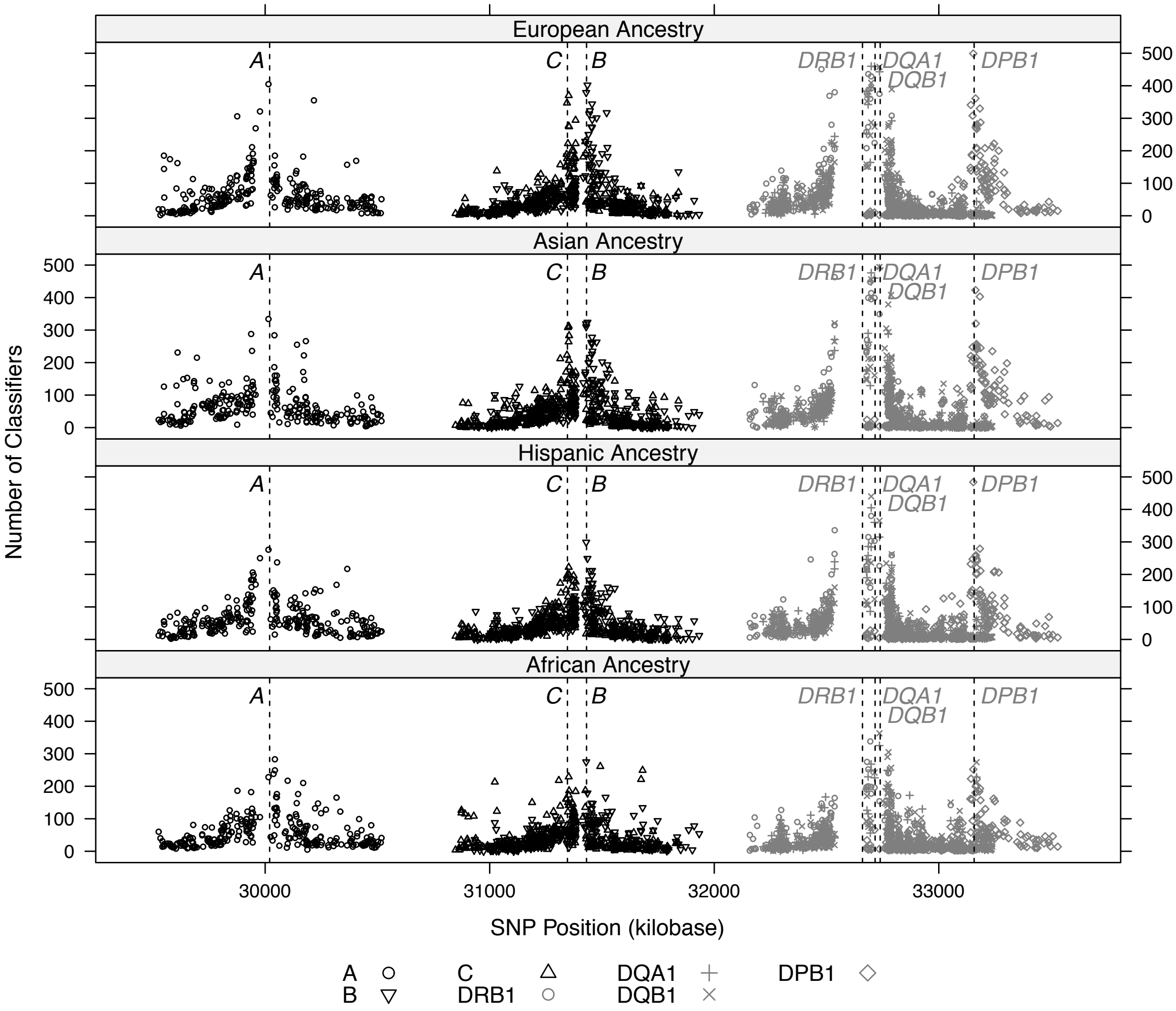

Figure S8: The number of classifiers used in the published pre-fit models for each SNP predictor. Each HIBAG model consists of 500 individual classifiers, and more important SNP markers tend to be used more frequently.

Supplementary Materials – Tables

Table S1: Assessing the prediction accuracies using different model parameter settings, when STUDY Data of European ancestry were divided into training and validation sets with equal sizes. No call threshold was executed[1].

	HLA –						
Accuracy (%)	*A*	*B*	*C*	*DRB1*	*DQA1*	*DQB1*	*DPB1*
# of SNPs [2]	273	341	356	327	349	356	279
# of training samples	945	1314	944	1234	874	968	820
# of validation samples	912	1258	922	1202	866	956	804
The total number of classifiers $K = 25$							
$m_{\text{try}} = 1$	98.1	95.9	98.3	91.4	96.4	97.4	93.3
$m_{\text{try}} = \sqrt{m}$	98.4	96.4	98.5	92.0	97.2	98.6	93.7
$m_{\text{try}} = \frac{1}{3}m$	98.2	95.7	99.0	91.3	96.7	98.6	93.5
$m_{\text{try}} = m$	98.5	95.7	99.0	91.5	96.7	98.5	93.5
The total number of classifiers $K = 100$							
$m_{\text{try}} = 1$	98.2	96.1	98.3	92.1	96.6	97.7	93.4
$m_{\text{try}} = \sqrt{m}$	98.2	96.6	98.8	92.1	97.3	98.8	93.8
$m_{\text{try}} = \frac{1}{3}m$	98.4	95.8	99.1	91.5	96.8	98.7	93.7
$m_{\text{try}} = m$	98.5	95.8	99.0	91.7	96.7	98.7	93.4

[1]: K is the total number of individual classifiers, m is the total number of SNP markers, and m_{try} is the number of variables randomly sampled as candidates for each selection.

[2]: SNP markers common to the Illumina 1M Duo, OmniQuad, OmniExpress, 660K and 550K platforms within a flanking region of 500kb are used.

Table S2: Assessing the computational times (hour) of building a HIBAG model for our published parameter estimates of European ancestry on a Linux system with Intel processor (2.27GHz) and 32 GB RAM.

	HLA –						
	A	*B*	*C*	*DRB1*	*DQA1*	*DQB1*	*DPB1*
# of SNPs [1]	273	341	356	327	349	356	279
# of HLA alleles	48	88	37	55	17	21	26
# of training samples	1504	2030	1493	1909	1380	1517	1274
Building a HIBAG model: computing time per individual classifier							
	0.86h	6.12h	0.84h	3.36h	0.58h	0.56h	0.28h

[1]: SNP markers common to the Illumina 1M Duo, OmniQuad, OmniExpress, 660K and 550K platforms within a flanking region of 500kb are used.

Table S3: Summary of the four-digit accuracies from HIBAG and BEAGLE using the same SNP sets. STUDY Data were randomly divided into training and validation sets with equal sizes for each ancestry. No call threshold was used, and the SNP markers within a 500kb flanking region on each side were used.

	HLA –						
Accuracy (%)	*A*	*B*	*C*	*DRB1*	*DQA1*	*DQB1*	*DPB1*
European ancestry							
# of training samples	945	1314	944	1234	874	968	820
# of validation samples	912	1258	922	1202	866	956	804
# of HLA alleles	48	88	37	55	17	21	26
# of SNPs[1] (1M/intersect)	937/273	942/341	979/356	921/327	964/349	979/356	786/279
BEAGLE – 1M	98.5	95.9	98.4	93.3	97.1	98.7	95.2
HIBAG – 1M	98.5	96.4	98.6	92.4	97.3	98.7	95.9
BEAGLE – Common	98.1	95.5	97.7	92.9	96.4	97.9	94.7
HIBAG – Common	98.2	96.6	98.8	92.1	97.3	98.8	93.8
Asian ancestry							
# of training samples	317	378	318	363	298	313	271
# of validation samples	289	335	293	333	286	299	256
# of HLA alleles	42	72	34	48	17	18	27
# of SNPs[1] (1M/intersect)	942/259	942/334	974/346	934/319	973/341	995/348	803/272
BEAGLE – 1M	93.4	87.0	96.6	89.5	87.0	98.2	91.8
HIBAG – 1M	92.1	87.8	96.9	91.7	89.2	98.2	91.0
BEAGLE – Common	93.8	83.7	94.5	87.7	86.7	97.3	91.2
HIBAG – Common	92.1	87.5	96.6	88.7	86.8	96.0	89.8
Hispanic ancestry							
# of training samples	161	238	157	223	139	162	139
# of validation samples	137	192	143	197	130	150	124
# of HLA alleles	41	85	32	44	14	17	26
# of SNPs[1] (1M/intersect)	965/274	966/341	996/356	954/326	992/348	1013/355	824/278
BEAGLE – 1M	88.7	75.8	92.0	78.4	94.2	97.7	94.9
HIBAG – 1M	91.6	74.0	95.5	82.0	96.9	98.0	95.6
BEAGLE – Common	89.1	75.0	92.3	78.7	94.6	96.3	91.9
HIBAG – Common	93.4	75.0	96.2	82.0	93.8	95.7	93.1
African ancestry							
# of training samples	81	100	74	89	69	74	44
# of validation samples	59	71	65	72	65	63	31
# of HLA alleles	36	45	24	30	13	17	23
# of SNPs[1] (1M/intersect)	949/266	948/335	981/349	945/325	983/343	1004/351	816/269
BEAGLE – 1M	96.6	70.4	87.7	73.6	86.2	84.1	85.5
HIBAG – 1M	95.8	81.0	90.0	84.0	85.4	80.2	82.3
BEAGLE – Common	93.2	71.1	86.9	81.2	79.2	76.2	79.0
HIBAG – Common	92.4	76.8	88.5	77.1	80.0	79.4	74.2

[1]: Illumina Human1M / Common to the Illumina 1M Duo, OmniQuad, OmniExpress, 660K and 550K platforms.

Table S4: The SNP list used by HLA*IMP when Illumina 1M platform is specified.

Locus (# of SNPs)	Marker list
HLA–A (50)	rs1737083, rs9391630, rs4947236, rs9258275, rs1737060, rs1633041, rs1737043, rs1633021, rs9258437, rs2517922, rs1632988, rs2523409, rs1610663, rs1610707, rs3115630, rs915669, rs2517892, rs2734999, rs3115629, rs2394185, rs9380146, rs9258631, rs2734979, rs2508049, rs2508046, rs3094159, rs2734959, rs2517817, rs2517904, rs2517891, rs1611493, rs2517755, rs2860580, rs2517722, rs7745413, rs7747253, rs7739434, rs9260759, rs5009448, rs2735076, rs2735071, rs3132685, rs5025708, rs3121597, rs166326, rs16896944, rs3132129, rs1150738, rs1245371, rs11965797
HLA–B (39)	rs1265156, rs4713438, rs3130466, rs9295967, rs9263979, rs6904669, rs2524099, rs2524123, rs9461688, rs2524229, rs9295976, rs9265797, rs2442719, rs2596501, rs1058026, rs2523591, rs2523590, rs2523589, rs2523578, rs2523557, rs2844573, rs6936035, rs3094600, rs9266689, rs2442752, rs2596560, rs2523471, rs2256175, rs9266845, rs3094738, rs2596460, rs3094228, rs2395030, rs2284178, rs2523674, rs2905722, rs2523710, rs2534671, rs2855807
HLA–C (27)	rs9263719, rs3823417, rs1265099, rs2074478, rs1265094, rs130075, rs9263800, rs1265158, rs3130467, rs3130531, rs2844623, rs9264532, rs2524099, rs2395471, rs2249742, rs2524084, rs12111032, rs6906846, rs6917363, rs3915971, rs7761965, rs9264942, rs7453967, rs2442719, rs2523535, rs2844529, rs3763288
HLA–DRB1 (50)	rs6907322, rs2273017, rs2050190, rs2076536, rs2073045, rs2050189, rs4248166, rs3817964, rs3793126, rs10947262, rs3806155, rs6932542, rs2395163, rs3135363, rs2027856, rs3129882, rs2239804, rs6919855, rs9268862, rs5020946, rs9270623, rs4599680, rs615672, rs2858867, rs482044, rs660895, rs2097431, rs9273012, rs6906021, rs3134975, rs9275184, rs7774434, rs2647044, rs9275424, rs9275425, rs9275572, rs7745656, rs2858332, rs3957148, rs3873444, rs9275602, rs3892710, rs5024431, rs12177980, rs7755596, rs17500468, rs10807113, rs4947347, rs2071550, rs6903130
HLA–DQB1 (34)	rs3117099, rs3817964, rs3763305, rs743862, rs3129867, rs2239802, rs4599680, rs482044, rs13207945, rs9272723, rs6927022, rs6906021, rs1063355, rs2300825, rs3891175, rs3828796, rs7755224, rs3134975, rs2856691, rs2856683, rs7774434, rs9275224, rs9275313, rs3135006, rs9275555, rs3104402, rs3104405, rs9275601, rs3892710, rs6935940, rs2395246, rs17500510, rs2071800, rs719654

Table S5: The sensitivity (SEN), specificity (SPE), positive predictive value (PPV) and negative predictive value (NPV) calculated from validation samples for each four-digit HLA allele with call threshold 0.5, when STUDY Data of European ancestry were divided to training and validation parts with equal sizes. The SNP markers in the intersect of Illumina platforms were used.

Allele[1]	Num. Train	Freq. Train	Num. Valid.	Freq. Valid.	CR[2] (%)	ACC[3] (%)	SEN (%)	SPE (%)	PPV (%)	NPV (%)	Miscall[4] (%)
HLA–A: Overall accuracy: 98.7%											
01:01	270	0.1429	271	0.1486	99.6	100.0	100.0	100.0	100.0	100.0	–
02:01	503	0.2661	533	0.2922	99.2	99.7	100.0	99.5	98.9	100.0	–
02:02	3	0.0016	3	0.0016	100.0	99.9	66.7	100.0	100.0	99.9	02:05 (100)
02:05	19	0.0101	19	0.0104	100.0	99.9	100.0	99.9	95.0	100.0	–
03:01	252	0.1333	234	0.1283	99.6	99.8	100.0	99.8	98.7	100.0	–
11:01	127	0.0672	120	0.0658	99.2	100.0	100.0	100.0	100.0	100.0	–
23:01	39	0.0206	42	0.0230	100.0	100.0	100.0	100.0	100.0	100.0	–
24:02	170	0.0899	141	0.0773	97.9	99.7	100.0	99.7	96.5	100.0	–
25:01	55	0.0291	50	0.0274	98.0	99.6	91.8	99.8	91.8	99.8	26:01 (100)
26:01	74	0.0392	70	0.0384	98.6	99.4	92.8	99.7	92.8	99.7	25:01 (80)
29:01	10	0.0053	10	0.0055	80.0	100.0	100.0	100.0	100.0	100.0	–
29:02	63	0.0333	57	0.0312	98.2	100.0	100.0	100.0	100.0	100.0	–
30:01	30	0.0159	27	0.0148	100.0	100.0	100.0	100.0	100.0	100.0	–
30:02	19	0.0101	17	0.0093	100.0	100.0	100.0	100.0	100.0	100.0	–
31:01	46	0.0243	45	0.0247	93.3	100.0	100.0	100.0	100.0	100.0	–
32:01	69	0.0365	66	0.0362	100.0	100.0	100.0	100.0	100.0	100.0	–
33:01	13	0.0069	12	0.0066	100.0	100.0	100.0	100.0	100.0	100.0	–
33:03	9	0.0048	9	0.0049	100.0	100.0	100.0	100.0	100.0	100.0	–
34:02	2	0.0011	1	0.0005	100.0	100.0	100.0	100.0	100.0	100.0	–
66:01	12	0.0063	11	0.0060	100.0	99.9	90.9	100.0	100.0	99.9	26:01 (100)
68:01	54	0.0286	58	0.0318	96.6	100.0	100.0	100.0	100.0	100.0	–
68:02	13	0.0069	11	0.0060	100.0	100.0	100.0	100.0	100.0	100.0	–
69:01	2	0.0011	2	0.0011	50.0	100.0	100.0	100.0	100.0	100.0	–
HLA–B: Overall accuracy: 97.8%											
07:02	319	0.1214	291	0.1157	97.3	99.9	100.0	99.9	99.3	100.0	–
07:05	8	0.0030	7	0.0028	100.0	100.0	100.0	100.0	100.0	100.0	–
08:01	239	0.0909	262	0.1041	97.3	100.0	100.0	100.0	100.0	100.0	–
13:02	87	0.0331	85	0.0338	94.1	100.0	100.0	100.0	100.0	100.0	–
14:01	17	0.0065	17	0.0068	82.4	100.0	100.0	100.0	100.0	100.0	–
14:02	61	0.0232	60	0.0238	96.7	100.0	100.0	100.0	100.0	100.0	–
15:01	147	0.0559	160	0.0636	98.1	99.8	100.0	99.8	97.5	100.0	–
15:17	10	0.0038	10	0.0040	100.0	100.0	100.0	100.0	100.0	100.0	–
15:18	5	0.0019	4	0.0016	100.0	100.0	75.0	100.0	100.0	100.0	15:01 (100)
18:01	140	0.0533	152	0.0604	93.4	99.6	99.3	99.6	94.6	100.0	38:01 (100)
27:02	24	0.0091	23	0.0091	95.7	99.7	72.7	100.0	100.0	99.8	27:05 (83)
27:05	98	0.0373	95	0.0378	96.8	99.8	100.0	99.8	94.8	100.0	–
35:01	149	0.0567	125	0.0497	88.0	99.4	95.5	99.6	91.3	99.8	35:02 (60)
35:02	24	0.0091	24	0.0095	83.3	99.9	100.0	99.9	87.0	100.0	–
35:03	54	0.0205	61	0.0242	91.8	99.6	82.1	100.0	100.0	99.6	35:01 (80)
35:08	19	0.0072	18	0.0072	55.6	99.9	90.0	99.9	81.8	100.0	35:01 (100)

Continued on next page ...

Table S5 – continued from previous page

Allele[1]	Num. Train	Freq. Train	Num. Valid.	Freq. Valid.	CR[2] (%)	ACC[3] (%)	SEN (%)	SPE (%)	PPV (%)	NPV (%)	Miscall[4] (%)
37:01	28	0.0107	26	0.0103	100.0	100.0	100.0	100.0	100.0	100.0	–
38:01	70	0.0266	74	0.0294	82.4	100.0	100.0	100.0	98.4	100.0	–
39:01	38	0.0145	35	0.0139	80.0	99.9	89.3	100.0	100.0	99.9	18:01 (67)
39:06	12	0.0046	11	0.0044	63.6	100.0	100.0	100.0	100.0	100.0	–
39:24	2	0.0008	1	0.0004	100.0	100.0	100.0	100.0	100.0	100.0	–
40:01	105	0.0400	98	0.0390	98.0	100.0	100.0	100.0	100.0	100.0	–
40:02	42	0.0160	47	0.0187	87.2	100.0	100.0	100.0	100.0	100.0	–
40:06	3	0.0011	3	0.0012	66.7	100.0	100.0	100.0	100.0	100.0	–
41:01	12	0.0046	11	0.0044	90.9	100.0	100.0	100.0	100.0	100.0	–
41:02	17	0.0065	15	0.0060	93.3	100.0	100.0	100.0	100.0	100.0	–
44:02	226	0.0860	212	0.0843	99.1	99.9	99.0	100.0	99.5	99.9	44:03 (50)
44:03	126	0.0479	126	0.0501	96.8	99.9	100.0	99.9	98.4	100.0	–
44:05	16	0.0061	13	0.0052	84.6	99.9	100.0	99.9	84.6	100.0	–
45:01	13	0.0049	12	0.0048	83.3	100.0	100.0	100.0	100.0	100.0	–
47:01	7	0.0027	7	0.0028	100.0	100.0	100.0	100.0	100.0	100.0	–
48:01	3	0.0011	2	0.0008	100.0	100.0	100.0	100.0	100.0	100.0	–
49:01	44	0.0167	44	0.0175	95.5	99.9	95.2	100.0	100.0	99.9	44:03 (50)
50:01	37	0.0141	35	0.0139	91.4	100.0	100.0	100.0	97.0	100.0	–
51:01	154	0.0586	122	0.0485	97.5	99.7	99.2	99.7	95.2	100.0	44:05 (100)
52:01	34	0.0129	31	0.0123	87.1	100.0	100.0	100.0	100.0	100.0	–
53:01	7	0.0027	6	0.0024	100.0	100.0	83.3	100.0	100.0	100.0	35:01 (100)
55:01	42	0.0160	44	0.0175	95.5	99.8	92.9	100.0	97.5	99.9	56:01 (100)
56:01	25	0.0095	21	0.0083	95.2	99.8	95.0	99.8	82.6	100.0	55:01 (100)
57:01	76	0.0289	77	0.0306	97.4	100.0	100.0	100.0	100.0	100.0	–
58:01	25	0.0095	22	0.0087	95.5	100.0	100.0	100.0	100.0	100.0	–
73:01	3	0.0011	2	0.0008	100.0	100.0	100.0	100.0	100.0	100.0	–
HLA–C: Overall accuracy: 99.2%											
01:02	85	0.0450	72	0.0390	98.6	100.0	100.0	100.0	100.0	100.0	–
02:02	115	0.0609	93	0.0504	100.0	100.0	100.0	100.0	100.0	100.0	–
03:02	4	0.0021	4	0.0022	100.0	100.0	100.0	100.0	100.0	100.0	–
03:03	90	0.0477	92	0.0499	91.3	99.7	97.6	99.8	95.3	99.9	03:04 (100)
03:04	113	0.0599	106	0.0575	97.2	99.7	96.1	99.9	98.0	99.8	03:03 (100)
04:01	221	0.1171	206	0.1117	99.5	100.0	100.0	100.0	100.0	100.0	–
05:01	143	0.0757	157	0.0851	99.4	99.9	99.4	99.9	99.4	99.9	08:02 (100)
06:02	176	0.0932	174	0.0944	99.4	99.9	100.0	99.9	99.4	100.0	–
07:01	278	0.1472	273	0.1480	97.8	100.0	100.0	100.0	100.0	100.0	–
07:02	228	0.1208	257	0.1394	99.2	100.0	100.0	100.0	100.0	100.0	–
07:04	38	0.0201	37	0.0201	100.0	100.0	100.0	100.0	100.0	100.0	–
08:02	48	0.0254	49	0.0266	98.0	99.9	97.9	99.9	97.9	99.9	05:01 (100)
08:03	2	0.0011	1	0.0005	100.0	100.0	100.0	100.0	100.0	100.0	–
12:02	26	0.0138	24	0.0130	95.8	100.0	100.0	100.0	100.0	100.0	–
12:03	134	0.0710	128	0.0694	99.2	99.9	99.2	100.0	100.0	99.9	06:02 (100)
14:02	21	0.0111	21	0.0114	100.0	100.0	100.0	100.0	100.0	100.0	–
15:02	43	0.0228	45	0.0244	93.3	99.9	100.0	99.9	95.5	100.0	–
15:05	8	0.0042	8	0.0043	100.0	100.0	100.0	100.0	100.0	100.0	–
16:01	63	0.0334	62	0.0336	96.8	99.9	100.0	99.9	98.4	100.0	–

Continued on next page ...

Table S5 – continued from previous page

Allele[1]	Num. Train	Freq. Train	Num. Valid.	Freq. Valid.	CR[2] (%)	ACC[3] (%)	SEN (%)	SPE (%)	PPV (%)	NPV (%)	Miscall[4] (%)
16:02	6	0.0032	5	0.0027	100.0	100.0	100.0	100.0	100.0	100.0	–
16:04	5	0.0026	4	0.0022	75.0	99.9	66.7	100.0	100.0	99.9	16:01 (100)
17:01	22	0.0117	18	0.0098	94.4	99.9	100.0	99.9	89.5	100.0	–
HLA–DRB1: Overall accuracy: 94.9%											
01:01	198	0.0802	208	0.0865	97.6	99.4	99.5	99.4	94.4	100.0	01:02 (100)
01:02	33	0.0134	35	0.0146	97.1	100.0	100.0	100.0	97.1	100.0	–
01:03	18	0.0073	18	0.0075	83.3	99.4	20.0	100.0	100.0	99.5	01:01 (100)
03:01	277	0.1122	277	0.1152	94.9	99.9	99.6	99.9	99.6	100.0	15:01 (100)
04:01	179	0.0725	198	0.0824	91.9	99.2	97.3	99.3	93.2	99.8	04:07 (80)
04:02	29	0.0118	28	0.0116	82.1	99.9	100.0	99.9	92.0	100.0	–
04:03	25	0.0101	31	0.0129	64.5	99.2	15.0	100.0	100.0	99.3	04:04 (65)
04:04	74	0.0300	64	0.0266	85.9	99.2	94.5	99.3	78.2	99.9	04:01 (100)
04:05	17	0.0069	19	0.0079	57.9	100.0	90.9	100.0	100.0	100.0	04:01 (100)
04:07	25	0.0101	28	0.0116	71.4	99.5	70.0	99.8	77.8	99.7	04:01 (67)
07:01	343	0.1390	300	0.1248	94.7	99.9	99.6	99.9	99.6	100.0	04:01 (100)
08:01	65	0.0263	56	0.0233	98.2	100.0	100.0	100.0	99.1	100.0	–
08:03	4	0.0016	3	0.0012	66.7	100.0	100.0	100.0	100.0	100.0	–
08:04	5	0.0020	4	0.0017	50.0	100.0	100.0	100.0	100.0	100.0	–
09:01	26	0.0105	22	0.0092	90.9	100.0	100.0	100.0	100.0	100.0	–
10:01	16	0.0065	17	0.0071	82.4	100.0	100.0	100.0	100.0	100.0	–
11:01	165	0.0669	177	0.0736	75.7	97.7	99.3	97.6	73.5	100.0	12:01 (100)
11:02	6	0.0024	6	0.0025	50.0	100.0	66.7	100.0	100.0	100.0	11:01 (100)
11:04	110	0.0446	96	0.0399	78.1	98.7	62.7	100.0	100.0	98.8	11:01 (93)
12:01	35	0.0142	39	0.0162	87.2	99.9	94.1	100.0	97.0	99.9	11:01 (100)
12:02	2	0.0008	1	0.0004	100.0	100.0	100.0	100.0	100.0	100.0	–
13:01	170	0.0689	142	0.0591	95.1	99.8	98.5	99.9	98.5	99.9	13:02 (50)
13:02	89	0.0361	87	0.0362	90.8	100.0	100.0	100.0	98.8	100.0	–
13:03	30	0.0122	31	0.0129	93.5	100.0	100.0	100.0	100.0	100.0	–
14:01	68	0.0276	67	0.0279	94.0	99.8	98.4	99.8	93.9	100.0	15:01 (100)
15:01	294	0.1191	300	0.1248	96.0	99.8	99.7	99.8	99.0	100.0	03:01 (100)
15:02	28	0.0113	28	0.0116	96.4	100.0	100.0	100.0	100.0	100.0	–
16:01	68	0.0276	68	0.0283	92.6	99.9	100.0	99.9	96.9	100.0	–
16:02	7	0.0028	7	0.0029	85.7	100.0	83.3	100.0	100.0	100.0	16:01 (100)
HLA–DQA1: Overall accuracy: 97.8%											
01:01	202	0.1156	185	0.1068	99.5	99.7	97.3	100.0	100.0	99.7	01:04 (80)
01:02	351	0.2008	355	0.2050	98.0	99.8	99.7	99.9	99.4	99.9	01:03 (100)
01:03	147	0.0841	138	0.0797	95.7	99.8	98.5	99.9	99.2	99.9	01:02 (100)
01:04	30	0.0172	29	0.0167	96.6	99.7	100.0	99.7	84.8	100.0	–
01:05	6	0.0034	6	0.0035	66.7	99.9	100.0	99.9	88.9	100.0	–
02:01	237	0.1356	263	0.1518	98.9	100.0	100.0	100.0	100.0	100.0	–
03:01	164	0.0938	159	0.0918	98.1	98.6	94.2	99.1	91.3	99.4	03:03 (89)
03:02	14	0.0080	14	0.0081	85.7	100.0	100.0	100.0	100.0	100.0	–
03:03	108	0.0618	108	0.0624	98.1	98.7	86.8	99.5	91.5	99.1	03:01 (100)
04:01	54	0.0309	52	0.0300	94.2	99.8	100.0	99.8	94.2	100.0	–
05:01	194	0.1110	183	0.1057	100.0	100.0	100.0	100.0	100.0	100.0	–
05:05	227	0.1299	228	0.1316	98.7	99.8	100.0	99.7	98.3	100.0	–

Continued on next page ...

Table S5 – continued from previous page

Allele[1]	Num. Train	Freq. Train	Num. Valid.	Freq. Valid.	CR[2] (%)	ACC[3] (%)	SEN (%)	SPE (%)	PPV (%)	NPV (%)	Miscall[4] (%)
06:01	5	0.0029	5	0.0029	80.0	99.9	50.0	100.0	100.0	99.9	04:01 (100)
HLA–DQB1 Overall accuracy: 99.2%											
02:01	220	0.1136	204	0.1067	99.0	100.0	100.0	100.0	100.0	100.0	–
02:02	200	0.1033	200	0.1046	98.0	99.9	100.0	99.9	99.5	100.0	–
03:01	393	0.2030	384	0.2008	98.7	99.7	99.7	99.7	98.8	99.9	02:02 (100)
03:02	187	0.0966	187	0.0978	98.9	99.9	100.0	99.9	98.9	100.0	–
03:03	84	0.0434	83	0.0434	94.0	99.9	98.7	100.0	100.0	99.9	03:01 (100)
03:04	5	0.0026	5	0.0026	60.0	99.9	33.3	100.0	100.0	99.9	03:01 (100)
03:19	2	0.0010	2	0.0010	100.0	99.9	50.0	100.0	100.0	99.9	03:01 (100)
04:02	61	0.0315	60	0.0314	98.3	100.0	100.0	100.0	100.0	100.0	–
05:01	220	0.1136	213	0.1114	98.6	99.9	99.0	100.0	100.0	99.9	05:03 (100)
05:02	67	0.0346	65	0.0340	98.5	100.0	100.0	100.0	100.0	100.0	–
05:03	55	0.0284	56	0.0293	100.0	99.9	100.0	99.9	96.6	100.0	–
06:01	24	0.0124	22	0.0115	95.5	100.0	100.0	100.0	100.0	100.0	–
06:02	234	0.1209	228	0.1192	97.8	99.7	99.6	99.8	98.4	99.9	06:04 (100)
06:03	112	0.0579	132	0.0690	97.0	99.8	97.7	100.0	100.0	99.8	06:02 (100)
06:04	52	0.0269	55	0.0288	94.5	99.8	98.1	99.9	96.2	99.9	03:01 (50)
06:09	14	0.0072	13	0.0068	92.3	99.9	91.7	100.0	100.0	99.9	06:04 (100)
HLA–DPB1: Overall accuracy: 94.8%											
01:01	74	0.0451	77	0.0479	96.1	100.0	100.0	100.0	100.0	100.0	–
02:01	201	0.1226	233	0.1449	96.6	98.0	90.7	99.2	95.3	98.5	04:01 (95)
03:01	133	0.0811	131	0.0815	93.9	98.1	99.2	98.0	80.8	99.9	104:01 (100)
04:01	713	0.4348	678	0.4216	98.5	97.8	99.7	96.3	95.3	99.8	02:01 (100)
04:02	212	0.1293	201	0.1250	97.0	99.5	100.0	99.5	96.8	100.0	–
05:01	33	0.0201	32	0.0199	100.0	100.0	100.0	100.0	100.0	100.0	–
09:01	12	0.0073	10	0.0062	50.0	99.9	100.0	99.9	83.3	100.0	–
10:01	22	0.0134	24	0.0149	91.7	99.9	95.5	100.0	100.0	99.9	09:01 (100)
11:01	29	0.0177	25	0.0155	88.0	100.0	100.0	100.0	100.0	100.0	–
13:01	38	0.0232	33	0.0205	87.9	100.0	100.0	100.0	100.0	100.0	–
14:01	20	0.0122	21	0.0131	66.7	100.0	100.0	100.0	100.0	100.0	–
15:01	16	0.0098	15	0.0093	100.0	100.0	100.0	100.0	100.0	100.0	–
16:01	7	0.0043	6	0.0037	100.0	100.0	100.0	100.0	100.0	100.0	–
17:01	29	0.0177	31	0.0193	93.5	100.0	100.0	100.0	100.0	100.0	–
19:01	8	0.0049	8	0.0050	100.0	100.0	100.0	100.0	100.0	100.0	–
104:01	24	0.0146	24	0.0149	87.5	99.9	100.0	99.9	91.3	100.0	–

[1]: the HLA alleles with more than one copy and non-zero sensitivity in the training are listed.
[2]: CR – call rate.
[3]: ACC – allele accuracy.
[4]: the most likely miscalled allele and the proportion of the most likely miscalled allele in all miscalled alleles.

Table S6: The sensitivity (SEN), specificity (SPE), positive predictive value (PPV) and negative predictive value (NPV) calculated from validation samples for each four-digit HLA allele with call threshold 0.5, when STUDY Data of Asian ancestry were divided to training and validation parts with equal sizes. The SNP markers in the intersect of Illumina platforms were used.

Allele[1]	Num. Train	Freq. Train	Num. Valid.	Freq. Valid.	CR[2] (%)	ACC[3] (%)	SEN (%)	SPE (%)	PPV (%)	NPV (%)	Miscall[4] (%)
HLA–A: Overall accuracy: 93.8%											
01:01	21	0.0331	21	0.0363	95.2	100.0	100.0	100.0	100.0	100.0	–
02:01	81	0.1278	65	0.1125	84.6	97.4	94.5	97.7	82.5	99.4	02:07 (67)
02:05	2	0.0032	2	0.0035	100.0	100.0	100.0	100.0	100.0	100.0	–
02:06	39	0.0615	27	0.0467	92.6	99.6	100.0	99.6	92.6	100.0	–
02:07	31	0.0489	32	0.0554	81.2	99.2	92.3	99.6	92.3	99.6	02:01 (100)
02:11	9	0.0142	8	0.0138	87.5	100.0	100.0	100.0	100.0	100.0	–
03:01	16	0.0252	13	0.0225	100.0	100.0	100.0	100.0	100.0	100.0	–
03:02	3	0.0047	2	0.0035	50.0	100.0	100.0	100.0	100.0	100.0	–
11:01	112	0.1767	120	0.2076	92.5	99.6	99.1	99.8	99.1	99.8	11:02 (100)
11:02	10	0.0158	7	0.0121	85.7	99.8	100.0	99.8	85.7	100.0	–
24:02	102	0.1609	103	0.1782	94.2	97.9	100.0	97.5	89.8	100.0	–
26:01	23	0.0363	17	0.0294	100.0	99.2	100.0	99.2	81.0	100.0	–
29:01	2	0.0032	1	0.0017	100.0	100.0	100.0	100.0	100.0	100.0	–
30:01	22	0.0347	20	0.0346	100.0	100.0	100.0	100.0	100.0	100.0	–
30:04	4	0.0063	3	0.0052	100.0	100.0	100.0	100.0	100.0	100.0	–
31:01	24	0.0379	21	0.0363	100.0	100.0	100.0	100.0	100.0	100.0	–
32:01	8	0.0126	9	0.0156	88.9	100.0	100.0	100.0	100.0	100.0	–
33:03	58	0.0915	59	0.1021	98.3	100.0	100.0	100.0	100.0	100.0	–
34:01	9	0.0142	7	0.0121	85.7	99.8	100.0	99.8	85.7	100.0	–
68:01	10	0.0158	7	0.0121	85.7	100.0	100.0	100.0	100.0	100.0	–
HLA–B: Overall accuracy: 94.7%											
07:02	22	0.0291	16	0.0239	93.8	100.0	100.0	100.0	100.0	100.0	–
07:05	12	0.0159	9	0.0134	88.9	100.0	100.0	100.0	100.0	100.0	–
08:01	11	0.0146	11	0.0164	90.9	100.0	100.0	100.0	100.0	100.0	–
13:01	27	0.0357	22	0.0328	77.3	100.0	100.0	100.0	100.0	100.0	–
13:02	27	0.0357	26	0.0388	84.6	100.0	100.0	100.0	100.0	100.0	–
14:01	4	0.0053	3	0.0045	66.7	100.0	100.0	100.0	100.0	100.0	–
15:01	24	0.0317	28	0.0418	46.4	99.0	92.3	99.2	75.0	99.8	46:01 (100)
15:02	22	0.0291	19	0.0284	84.2	99.8	93.8	100.0	100.0	99.8	15:35 (100)
15:17	3	0.0040	4	0.0060	50.0	100.0	100.0	100.0	100.0	100.0	–
15:18	10	0.0132	9	0.0134	77.8	100.0	100.0	100.0	100.0	100.0	–
15:35	6	0.0079	3	0.0045	100.0	99.8	100.0	99.8	75.0	100.0	–
18:01	8	0.0106	7	0.0104	42.9	100.0	100.0	100.0	100.0	100.0	–
27:04	8	0.0106	4	0.0060	100.0	99.6	100.0	99.6	66.7	100.0	–
27:05	9	0.0119	9	0.0134	66.7	100.0	100.0	100.0	100.0	100.0	–
35:01	27	0.0357	24	0.0358	70.8	99.4	100.0	99.4	85.0	100.0	–
35:03	17	0.0225	11	0.0164	54.5	99.4	50.0	100.0	100.0	99.6	35:01 (100)
35:05	6	0.0079	6	0.0090	50.0	100.0	100.0	100.0	100.0	100.0	–
37:01	13	0.0172	10	0.0149	80.0	100.0	100.0	100.0	100.0	100.0	–
38:01	4	0.0053	3	0.0045	66.7	100.0	100.0	100.0	100.0	100.0	–

Continued on next page ...

Table S6 – continued from previous page

Allele[1]	Num. Train	Freq. Train	Num. Valid.	Freq. Valid.	CR[2] (%)	ACC[3] (%)	SEN (%)	SPE (%)	PPV (%)	NPV (%)	Miscall[4] (%)
38:02	23	0.0304	17	0.0254	82.4	99.2	100.0	99.1	77.8	100.0	–
39:01	11	0.0146	13	0.0194	53.8	99.4	57.1	100.0	100.0	99.5	38:02 (100)
40:01	61	0.0807	48	0.0716	85.4	100.0	100.0	100.0	100.0	100.0	–
40:02	22	0.0291	23	0.0343	65.2	99.2	86.7	99.6	86.7	99.7	40:06 (100)
40:06	37	0.0489	34	0.0507	67.6	99.0	91.3	99.3	87.5	99.7	27:04 (100)
44:02	7	0.0093	6	0.0090	66.7	100.0	100.0	100.0	100.0	100.0	–
44:03	29	0.0384	29	0.0433	89.7	100.0	100.0	100.0	100.0	100.0	–
46:01	46	0.0608	39	0.0582	84.6	99.6	97.0	99.8	97.0	99.8	40:02 (100)
47:01	2	0.0026	1	0.0015	100.0	100.0	100.0	100.0	100.0	100.0	–
48:01	13	0.0172	14	0.0209	64.3	99.8	100.0	99.8	90.0	100.0	–
50:01	4	0.0053	5	0.0075	80.0	100.0	100.0	100.0	100.0	100.0	–
51:01	50	0.0661	56	0.0836	83.9	99.2	95.7	99.5	95.7	99.7	51:02 (100)
51:02	4	0.0053	4	0.0060	75.0	99.4	66.7	99.6	50.0	99.8	51:01 (100)
51:06	3	0.0040	4	0.0060	75.0	100.0	100.0	100.0	100.0	100.0	–
52:01	31	0.0410	22	0.0328	68.2	99.8	93.3	100.0	100.0	99.8	51:01 (100)
54:01	27	0.0357	22	0.0328	72.7	99.4	93.8	99.6	88.2	99.8	55:02 (100)
57:01	10	0.0132	9	0.0134	88.9	100.0	100.0	100.0	100.0	100.0	–
58:01	35	0.0463	41	0.0612	90.2	100.0	100.0	100.0	100.0	100.0	–
67:01	4	0.0053	3	0.0045	66.7	99.8	50.0	100.0	100.0	99.9	38:02 (100)
HLA–C: Overall accuracy: 97.8%											
01:02	83	0.1305	73	0.1246	95.9	99.1	98.6	99.2	94.5	99.8	04:01 (50)
02:02	5	0.0079	4	0.0068	100.0	100.0	100.0	100.0	100.0	100.0	–
03:02	33	0.0519	29	0.0495	96.6	100.0	100.0	100.0	100.0	100.0	–
03:03	50	0.0786	38	0.0648	97.4	99.5	97.3	99.6	94.7	99.8	03:04 (100)
03:04	50	0.0786	53	0.0904	98.1	99.5	96.2	99.8	98.0	99.6	03:03 (100)
04:01	37	0.0582	32	0.0546	87.5	99.8	100.0	99.8	96.6	100.0	–
04:03	6	0.0094	5	0.0085	80.0	99.8	100.0	99.8	80.0	100.0	–
05:01	6	0.0094	6	0.0102	83.3	100.0	100.0	100.0	100.0	100.0	–
06:02	46	0.0723	45	0.0768	97.8	100.0	100.0	100.0	100.0	100.0	–
07:01	17	0.0267	14	0.0239	92.9	100.0	100.0	100.0	100.0	100.0	–
07:02	97	0.1525	104	0.1775	97.1	99.8	99.0	100.0	100.0	99.8	04:01 (50)
07:04	7	0.0110	6	0.0102	66.7	100.0	100.0	100.0	100.0	100.0	–
08:01	53	0.0833	50	0.0853	90.0	99.6	100.0	99.6	95.7	100.0	–
08:02	5	0.0079	5	0.0085	80.0	100.0	100.0	100.0	100.0	100.0	–
12:02	26	0.0409	25	0.0427	88.0	100.0	100.0	100.0	100.0	100.0	–
12:03	14	0.0220	11	0.0188	90.9	100.0	100.0	100.0	100.0	100.0	–
12:04	3	0.0047	3	0.0051	100.0	100.0	100.0	100.0	100.0	100.0	–
14:02	35	0.0550	32	0.0546	96.9	100.0	100.0	100.0	100.0	100.0	–
14:03	12	0.0189	13	0.0222	92.3	100.0	100.0	100.0	100.0	100.0	–
15:02	30	0.0472	30	0.0512	93.3	99.8	100.0	99.8	96.6	100.0	–
HLA–DRB1: Overall accuracy: 95.8%											
01:01	12	0.0165	14	0.0210	64.3	100.0	100.0	100.0	100.0	100.0	–
03:01	40	0.0551	34	0.0511	82.4	100.0	100.0	100.0	100.0	100.0	–
04:01	7	0.0096	7	0.0105	57.1	100.0	100.0	100.0	100.0	100.0	–
04:03	21	0.0289	20	0.0300	10.0	99.8	100.0	99.8	66.7	100.0	–
04:04	7	0.0096	6	0.0090	16.7	100.0	100.0	100.0	100.0	100.0	–

Continued on next page ...

Table S6 – continued from previous page

Allele[1]	Num. Train	Freq. Train	Num. Valid.	Freq. Valid.	CR[2] (%)	ACC[3] (%)	SEN (%)	SPE (%)	PPV (%)	NPV (%)	Miscall[4] (%)
04:05	42	0.0579	34	0.0511	70.6	99.6	91.7	100.0	100.0	99.7	04:10 (50)
04:06	17	0.0234	13	0.0195	30.8	100.0	100.0	100.0	100.0	100.0	–
04:10	5	0.0069	4	0.0060	25.0	99.8	100.0	99.8	50.0	100.0	–
07:01	65	0.0895	61	0.0916	83.6	100.0	100.0	100.0	100.0	100.0	–
08:02	10	0.0138	7	0.0105	85.7	99.6	100.0	99.6	75.0	100.0	–
08:03	36	0.0496	36	0.0541	69.4	99.8	100.0	99.8	96.2	100.0	–
09:01	86	0.1185	65	0.0976	90.8	100.0	100.0	100.0	100.0	100.0	–
10:01	16	0.0220	18	0.0270	66.7	100.0	100.0	100.0	100.0	100.0	–
11:01	35	0.0482	27	0.0405	70.4	99.1	100.0	99.1	82.6	100.0	–
12:01	22	0.0303	26	0.0390	57.7	99.4	80.0	100.0	100.0	99.5	11:01 (67)
12:02	40	0.0551	53	0.0796	88.7	99.8	100.0	99.8	97.9	100.0	–
13:01	16	0.0220	13	0.0195	69.2	100.0	100.0	100.0	100.0	100.0	–
13:02	31	0.0427	28	0.0420	78.6	100.0	100.0	100.0	100.0	100.0	–
13:12	3	0.0041	2	0.0030	50.0	100.0	100.0	100.0	100.0	100.0	–
14:01	12	0.0165	15	0.0225	20.0	99.4	33.3	99.8	50.0	99.7	14:04 (100)
14:03	6	0.0083	5	0.0075	100.0	99.8	80.0	100.0	100.0	99.8	11:01 (100)
14:04	15	0.0207	14	0.0210	64.3	99.4	100.0	99.3	75.0	100.0	–
14:05	15	0.0207	15	0.0225	46.7	99.8	85.7	100.0	100.0	99.8	14:04 (100)
15:01	70	0.0964	66	0.0991	77.3	99.8	98.0	100.0	100.0	99.8	16:02 (100)
15:02	48	0.0661	50	0.0751	82.0	100.0	100.0	100.0	100.0	100.0	–
16:02	12	0.0165	13	0.0195	46.2	99.8	100.0	99.8	85.7	100.0	–
HLA–DQA1: Overall accuracy: 90.0%											
01:01	34	0.0570	31	0.0542	83.9	98.7	76.9	100.0	100.0	98.9	01:04 (83)
01:02	109	0.1829	106	0.1853	85.8	98.9	96.7	99.5	97.8	99.4	01:03 (67)
01:03	73	0.1225	77	0.1346	87.0	99.4	98.5	99.5	97.1	99.8	01:02 (100)
01:04	28	0.0470	24	0.0420	58.3	98.9	100.0	98.9	73.7	100.0	–
01:05	8	0.0134	8	0.0140	75.0	100.0	100.0	100.0	100.0	100.0	–
02:01	54	0.0906	55	0.0962	94.5	100.0	100.0	100.0	100.0	100.0	–
03:01	83	0.1393	68	0.1189	76.5	95.3	61.5	99.5	94.1	96.3	03:02 (56)
03:02	50	0.0839	55	0.0962	65.5	97.4	97.2	97.4	75.7	99.8	03:03 (100)
03:03	28	0.0470	27	0.0472	59.3	97.4	87.5	97.8	58.9	99.6	03:01 (100)
04:01	9	0.0151	7	0.0122	100.0	100.0	100.0	100.0	100.0	100.0	–
05:01	34	0.0570	27	0.0472	92.6	98.9	80.0	100.0	100.0	99.1	05:05 (80)
05:03	6	0.0101	5	0.0087	20.0	99.8	100.0	99.8	50.0	100.0	–
05:05	29	0.0487	28	0.0490	85.7	98.7	100.0	98.6	80.0	100.0	–
05:08	7	0.0117	6	0.0105	83.3	99.8	80.0	100.0	100.0	99.8	05:05 (100)
06:01	39	0.0654	45	0.0787	91.1	99.8	100.0	99.8	97.6	100.0	–
HLA–DQB1 Overall accuracy: 98.1%											
02:01	25	0.0399	27	0.0452	100.0	100.0	100.0	100.0	100.0	100.0	–
02:02	44	0.0703	38	0.0635	100.0	99.8	97.4	100.0	100.0	99.8	03:03 (100)
03:01	111	0.1773	104	0.1739	99.0	98.6	98.1	98.7	94.4	99.6	03:03 (100)
03:02	45	0.0719	50	0.0836	92.0	100.0	100.0	100.0	100.0	100.0	–
03:03	83	0.1326	86	0.1438	96.5	99.0	96.4	99.4	96.4	99.4	03:01 (100)
04:01	32	0.0511	26	0.0435	96.2	99.7	96.0	99.8	96.0	99.8	04:02 (100)
04:02	13	0.0208	12	0.0201	100.0	99.7	91.7	99.8	91.7	99.8	04:01 (100)
05:01	36	0.0575	34	0.0569	100.0	100.0	100.0	100.0	100.0	100.0	–

Continued on next page ...

Table S6 – continued from previous page

Allele[1]	Num. Train	Freq. Train	Num. Valid.	Freq. Valid.	CR[2] (%)	ACC[3] (%)	SEN (%)	SPE (%)	PPV (%)	NPV (%)	Miscall[4] (%)
05:02	44	0.0703	38	0.0635	100.0	99.5	92.1	100.0	100.0	99.5	03:01 (100)
05:03	31	0.0495	35	0.0585	100.0	100.0	100.0	100.0	100.0	100.0	–
06:01	74	0.1182	78	0.1304	94.9	100.0	100.0	100.0	100.0	100.0	–
06:02	43	0.0687	34	0.0569	97.1	100.0	100.0	100.0	100.0	100.0	–
06:03	12	0.0192	12	0.0201	58.3	100.0	100.0	100.0	100.0	100.0	–
06:04	18	0.0288	13	0.0217	84.6	100.0	100.0	100.0	100.0	100.0	–
06:09	11	0.0176	11	0.0184	90.9	100.0	100.0	100.0	100.0	100.0	–
HLA–DPB1: Overall accuracy: 95.3%											
01:01	18	0.0332	15	0.0293	80.0	99.8	91.7	100.0	100.0	99.8	26:01 (100)
02:01	109	0.2011	113	0.2207	85.0	97.6	96.9	97.9	93.0	99.3	04:01 (67)
02:02	21	0.0387	24	0.0469	66.7	98.3	62.5	99.8	90.9	98.8	02:01 (100)
03:01	16	0.0295	13	0.0254	30.8	99.5	100.0	99.5	66.7	100.0	–
04:01	78	0.1439	66	0.1289	93.9	99.1	96.8	99.4	96.8	99.6	02:01 (50)
04:02	25	0.0461	29	0.0566	93.1	99.3	100.0	99.2	90.0	100.0	–
05:01	165	0.3044	154	0.3008	88.3	99.3	99.3	99.3	98.5	99.7	14:01 (100)
09:01	10	0.0185	12	0.0234	91.7	100.0	100.0	100.0	100.0	100.0	–
09:02	10	0.0185	8	0.0156	62.5	99.3	60.0	99.8	75.0	99.6	05:01 (50)
13:01	21	0.0387	20	0.0391	80.0	99.5	93.8	99.8	93.8	99.8	09:02 (100)
14:01	16	0.0295	18	0.0352	77.8	99.8	100.0	99.8	93.3	100.0	–
17:01	15	0.0277	14	0.0273	92.9	100.0	100.0	100.0	100.0	100.0	–
26:01	7	0.0129	6	0.0117	50.0	99.8	100.0	99.8	75.0	100.0	–
104:01	11	0.0203	9	0.0176	44.4	99.5	50.0	100.0	100.0	99.6	03:01 (100)

[1]: the HLA alleles with more than one copy and non-zero sensitivity in the training are listed.
[2]: CR – call rate.
[3]: ACC – allele accuracy.
[4]: the most likely miscalled allele and the proportion of the most likely miscalled allele in all miscalled alleles.

Table S7: The sensitivity (SEN), specificity (SPE), positive predictive value (PPV) and negative predictive value (NPV) calculated from validation samples for each four-digit HLA allele with call threshold 0.5, when STUDY Data of Hispanic ancestry were divided to training and validation parts with equal sizes. The SNP markers in the intersect of Illumina platforms were used.

Allele[1]	Num. Train	Freq. Train	Num. Valid.	Freq. Valid.	CR[2] (%)	ACC[3] (%)	SEN (%)	SPE (%)	PPV (%)	NPV (%)	Miscall[4] (%)
HLA–A: Overall accuracy: 96.0%											
01:01	22	0.0683	17	0.0620	100.0	100.0	100.0	100.0	100.0	100.0	–
02:01	78	0.2422	75	0.2737	84.0	98.2	100.0	97.5	94.0	100.0	–
02:05	2	0.0062	2	0.0073	50.0	100.0	100.0	100.0	100.0	100.0	–
02:06	4	0.0124	5	0.0182	40.0	100.0	100.0	100.0	100.0	100.0	–
03:01	21	0.0652	15	0.0547	100.0	100.0	100.0	100.0	100.0	100.0	–
11:01	14	0.0435	12	0.0438	83.3	100.0	100.0	100.0	100.0	100.0	–
23:01	13	0.0404	9	0.0328	88.9	100.0	100.0	100.0	100.0	100.0	–
24:02	33	0.1025	26	0.0949	80.8	100.0	100.0	100.0	100.0	100.0	–
26:01	13	0.0404	13	0.0474	84.6	99.1	100.0	99.1	84.6	100.0	–
29:02	17	0.0528	14	0.0511	100.0	100.0	100.0	100.0	100.0	100.0	–
30:01	6	0.0186	4	0.0146	75.0	100.0	100.0	100.0	100.0	100.0	–
30:02	5	0.0155	5	0.0182	60.0	100.0	100.0	100.0	100.0	100.0	–
30:04	2	0.0062	2	0.0073	100.0	100.0	100.0	100.0	100.0	100.0	–
31:01	20	0.0621	24	0.0876	91.7	100.0	100.0	100.0	100.0	100.0	–
32:01	6	0.0186	5	0.0182	80.0	100.0	100.0	100.0	100.0	100.0	–
33:01	4	0.0124	4	0.0146	75.0	100.0	100.0	100.0	100.0	100.0	–
68:01	18	0.0559	20	0.0730	70.0	98.7	92.9	99.1	86.7	99.6	68:17 (100)
68:02	4	0.0124	3	0.0109	100.0	100.0	100.0	100.0	100.0	100.0	–
68:17	4	0.0124	4	0.0146	25.0	99.6	100.0	99.6	50.0	100.0	–
HLA–B: Overall accuracy: 93.8%											
07:02	14	0.0294	16	0.0417	68.8	100.0	100.0	100.0	100.0	100.0	–
08:01	17	0.0357	12	0.0312	91.7	100.0	100.0	100.0	100.0	100.0	–
13:02	5	0.0105	10	0.0260	40.0	100.0	100.0	100.0	100.0	100.0	–
14:01	4	0.0084	3	0.0078	66.7	100.0	100.0	100.0	100.0	100.0	–
14:02	7	0.0147	9	0.0234	44.4	100.0	100.0	100.0	100.0	100.0	–
15:01	14	0.0294	9	0.0234	33.3	98.6	66.7	99.3	66.7	99.7	35:43 (100)
15:04	12	0.0252	14	0.0365	35.7	100.0	100.0	100.0	100.0	100.0	–
18:01	20	0.0420	15	0.0391	46.7	100.0	100.0	100.0	100.0	100.0	–
27:05	5	0.0105	3	0.0078	66.7	100.0	100.0	100.0	100.0	100.0	–
35:02	5	0.0105	4	0.0104	75.0	100.0	100.0	100.0	100.0	100.0	–
35:05	11	0.0231	11	0.0286	18.2	100.0	100.0	100.0	100.0	100.0	–
35:19	4	0.0084	2	0.0052	50.0	100.0	100.0	100.0	100.0	100.0	–
35:43	8	0.0168	5	0.0130	40.0	98.6	100.0	98.6	50.0	100.0	–
37:01	2	0.0042	3	0.0078	33.3	100.0	100.0	100.0	100.0	100.0	–
38:01	9	0.0189	7	0.0182	57.1	100.0	100.0	100.0	100.0	100.0	–
39:06	7	0.0147	3	0.0078	33.3	100.0	100.0	100.0	100.0	100.0	–
39:09	11	0.0231	6	0.0156	50.0	100.0	100.0	100.0	100.0	100.0	–
40:01	9	0.0189	5	0.0130	20.0	99.3	100.0	99.3	50.0	100.0	–
41:01	3	0.0063	2	0.0052	50.0	100.0	100.0	100.0	100.0	100.0	–
42:01	2	0.0042	1	0.0026	100.0	100.0	100.0	100.0	100.0	100.0	–

Continued on next page ...

Table S7 – continued from previous page

Allele[1]	Num. Train	Freq. Train	Num. Valid.	Freq. Valid.	CR[2] (%)	ACC[3] (%)	SEN (%)	SPE (%)	PPV (%)	NPV (%)	Miscall[4] (%)
44:02	10	0.0210	12	0.0312	50.0	99.3	83.3	100.0	100.0	99.7	40:01 (100)
44:03	36	0.0756	22	0.0573	77.3	100.0	100.0	100.0	100.0	100.0	–
48:01	19	0.0399	16	0.0417	50.0	100.0	100.0	100.0	100.0	100.0	–
49:01	11	0.0231	11	0.0286	54.5	100.0	100.0	100.0	100.0	100.0	–
50:01	5	0.0105	5	0.0130	60.0	100.0	100.0	100.0	100.0	100.0	–
51:01	37	0.0777	27	0.0703	48.1	99.3	100.0	99.2	92.9	100.0	–
52:01	11	0.0231	11	0.0286	72.7	99.3	87.5	100.0	100.0	99.7	35:01 (50)
53:01	4	0.0084	2	0.0052	50.0	100.0	100.0	100.0	100.0	100.0	–
57:01	8	0.0168	4	0.0104	100.0	100.0	100.0	100.0	100.0	100.0	–
58:01	5	0.0105	6	0.0156	50.0	100.0	100.0	100.0	100.0	100.0	–
HLA–C: Overall accuracy: 98.4%											
01:02	24	0.0764	25	0.0874	80.0	99.6	95.0	100.0	100.0	99.6	08:01 (100)
02:02	7	0.0223	6	0.0210	50.0	100.0	100.0	100.0	100.0	100.0	–
03:03	10	0.0318	5	0.0175	100.0	100.0	100.0	100.0	100.0	100.0	–
03:04	25	0.0796	26	0.0909	76.9	98.8	100.0	98.7	87.0	100.0	–
04:01	52	0.1656	49	0.1713	95.9	100.0	100.0	100.0	100.0	100.0	–
05:01	13	0.0414	12	0.0420	66.7	100.0	100.0	100.0	100.0	100.0	–
06:02	19	0.0605	17	0.0594	94.1	100.0	100.0	100.0	100.0	100.0	–
07:01	31	0.0987	29	0.1014	100.0	100.0	100.0	100.0	100.0	100.0	–
07:02	34	0.1083	30	0.1049	96.7	100.0	100.0	100.0	100.0	100.0	–
08:01	5	0.0159	5	0.0175	80.0	99.6	100.0	99.6	80.0	100.0	–
08:02	8	0.0255	8	0.0280	100.0	100.0	100.0	100.0	100.0	100.0	–
08:03	9	0.0287	7	0.0245	85.7	100.0	100.0	100.0	100.0	100.0	–
12:02	5	0.0159	5	0.0175	100.0	100.0	100.0	100.0	100.0	100.0	–
12:03	16	0.0510	12	0.0420	100.0	100.0	100.0	100.0	100.0	100.0	–
14:02	6	0.0191	6	0.0210	66.7	100.0	100.0	100.0	100.0	100.0	–
15:02	14	0.0446	15	0.0524	86.7	100.0	100.0	100.0	100.0	100.0	–
16:01	14	0.0446	14	0.0490	100.0	100.0	100.0	100.0	100.0	100.0	–
16:02	4	0.0127	3	0.0105	100.0	100.0	100.0	100.0	100.0	100.0	–
17:01	3	0.0096	4	0.0140	25.0	100.0	100.0	100.0	100.0	100.0	–
HLA–DRB1: Overall accuracy: 93.5%											
01:01	12	0.0269	12	0.0305	41.7	99.5	80.0	100.0	100.0	99.7	01:03 (100)
01:02	8	0.0179	9	0.0228	100.0	100.0	100.0	100.0	100.0	100.0	–
03:01	28	0.0628	27	0.0685	70.4	100.0	100.0	100.0	100.0	100.0	–
04:01	8	0.0179	6	0.0152	33.3	99.5	50.0	100.0	100.0	99.7	07:01 (50)
04:04	24	0.0538	16	0.0406	18.8	99.0	33.3	100.0	100.0	99.5	04:07 (100)
04:05	4	0.0090	4	0.0102	50.0	100.0	100.0	100.0	100.0	100.0	–
04:06	2	0.0045	2	0.0051	50.0	100.0	100.0	100.0	100.0	100.0	–
04:07	41	0.0919	31	0.0787	22.6	98.5	100.0	98.4	70.0	100.0	–
07:01	46	0.1031	42	0.1066	83.3	99.5	100.0	99.4	97.2	100.0	–
08:01	5	0.0112	5	0.0127	60.0	99.5	66.7	100.0	100.0	99.7	08:02 (100)
08:02	37	0.0830	32	0.0812	62.5	98.0	100.0	97.8	83.3	100.0	–
09:01	26	0.0583	24	0.0609	75.0	99.5	100.0	99.5	94.7	100.0	–
10:01	4	0.0090	3	0.0076	100.0	100.0	100.0	100.0	100.0	100.0	–
11:04	17	0.0381	17	0.0431	5.9	100.0	100.0	100.0	100.0	100.0	–
12:01	6	0.0135	6	0.0152	50.0	100.0	100.0	100.0	100.0	100.0	–

Continued on next page ...

Table S7 – continued from previous page

Allele[1]	Num. Train	Freq. Train	Num. Valid.	Freq. Valid.	CR[2] (%)	ACC[3] (%)	SEN (%)	SPE (%)	PPV (%)	NPV (%)	Miscall[4] (%)
13:01	14	0.0314	13	0.0330	69.2	100.0	100.0	100.0	100.0	100.0	–
13:02	16	0.0359	12	0.0305	50.0	100.0	100.0	100.0	100.0	100.0	–
14:01	6	0.0135	5	0.0127	80.0	100.0	100.0	100.0	100.0	100.0	–
14:02	22	0.0493	20	0.0508	55.0	98.5	81.8	99.5	90.0	99.5	14:06 (100)
14:06	9	0.0202	11	0.0279	63.6	98.5	85.7	99.0	75.0	99.7	07:01 (50)
15:01	20	0.0448	22	0.0558	68.2	100.0	100.0	100.0	100.0	100.0	–
15:02	6	0.0135	8	0.0203	87.5	100.0	100.0	100.0	100.0	100.0	–
16:01	3	0.0067	4	0.0102	100.0	100.0	100.0	100.0	100.0	100.0	–
16:02	9	0.0202	9	0.0228	11.1	100.0	100.0	100.0	100.0	100.0	–
HLA–DQA1: Overall accuracy: 95.8%											
01:01	20	0.0719	20	0.0769	100.0	98.3	100.0	98.1	83.3	100.0	–
01:02	28	0.1007	30	0.1154	83.3	98.7	92.0	99.5	95.8	99.2	01:01 (100)
01:03	14	0.0504	13	0.0500	92.3	99.6	91.7	100.0	100.0	99.6	01:02 (100)
01:04	3	0.0108	3	0.0115	66.7	99.6	50.0	100.0	100.0	99.6	01:01 (100)
02:01	37	0.1331	31	0.1192	100.0	100.0	100.0	100.0	100.0	100.0	–
03:01	57	0.2050	47	0.1808	89.4	97.9	100.0	97.4	89.4	100.0	–
03:02	15	0.0540	15	0.0577	86.7	99.2	84.6	100.0	100.0	99.2	03:01 (100)
03:03	11	0.0396	11	0.0423	72.7	98.7	62.5	100.0	100.0	98.8	03:01 (100)
04:01	33	0.1187	26	0.1000	100.0	100.0	100.0	100.0	100.0	100.0	–
05:01	21	0.0755	19	0.0731	100.0	100.0	100.0	100.0	100.0	100.0	–
05:03	11	0.0396	10	0.0385	90.0	100.0	100.0	100.0	100.0	100.0	–
05:05	26	0.0935	34	0.1308	82.4	100.0	100.0	100.0	100.0	100.0	–
HLA–DQB1 Overall accuracy: 98.9%											
02:01	23	0.0710	23	0.0767	91.3	100.0	100.0	100.0	100.0	100.0	–
02:02	36	0.1111	29	0.0967	96.6	99.6	96.4	100.0	100.0	99.6	03:01 (100)
03:01	62	0.1914	64	0.2133	89.1	98.9	100.0	98.6	95.0	100.0	–
03:02	62	0.1914	58	0.1933	96.6	100.0	100.0	100.0	100.0	100.0	–
03:03	20	0.0617	21	0.0700	76.2	100.0	100.0	100.0	100.0	100.0	–
04:02	39	0.1204	30	0.1000	90.0	100.0	100.0	100.0	100.0	100.0	–
05:01	27	0.0833	27	0.0900	100.0	100.0	100.0	100.0	100.0	100.0	–
05:03	4	0.0123	5	0.0167	60.0	100.0	100.0	100.0	100.0	100.0	–
06:01	5	0.0154	4	0.0133	100.0	100.0	100.0	100.0	100.0	100.0	–
06:02	19	0.0586	17	0.0567	82.4	100.0	100.0	100.0	100.0	100.0	–
06:03	11	0.0340	9	0.0300	100.0	100.0	100.0	100.0	100.0	100.0	–
06:04	7	0.0216	6	0.0200	100.0	100.0	100.0	100.0	100.0	100.0	–
HLA–DPB1: Overall accuracy: 97.5%											
01:01	10	0.0360	10	0.0403	100.0	100.0	100.0	100.0	100.0	100.0	–
02:01	31	0.1115	30	0.1210	70.0	99.0	95.2	99.4	95.2	99.6	04:01 (100)
02:02	3	0.0108	3	0.0121	66.7	100.0	100.0	100.0	100.0	100.0	–
04:01	70	0.2518	71	0.2863	87.3	99.5	100.0	99.3	98.4	100.0	–
04:02	75	0.2698	64	0.2581	96.9	100.0	100.0	100.0	100.0	100.0	–
05:01	10	0.0360	9	0.0363	88.9	100.0	100.0	100.0	100.0	100.0	–
11:01	8	0.0288	8	0.0323	75.0	100.0	100.0	100.0	100.0	100.0	–
13:01	13	0.0468	9	0.0363	77.8	99.5	100.0	99.5	87.5	100.0	–
14:01	18	0.0647	17	0.0685	88.2	99.5	100.0	99.5	93.8	100.0	–
17:01	5	0.0180	5	0.0202	40.0	100.0	100.0	100.0	100.0	100.0	–

Continued on next page ...

Table S7 – continued from previous page

Allele[1]	Num. Train	Freq. Train	Num. Valid.	Freq. Valid.	CR[2] (%)	ACC[3] (%)	SEN (%)	SPE (%)	PPV (%)	NPV (%)	Miscall[4] (%)
104:01	4	0.0144	4	0.0161	75.0	100.0	100.0	100.0	100.0	100.0	–

[1]: the HLA alleles with more than one copy and non-zero sensitivity in the training are listed.
[2]: CR – call rate.
[3]: ACC – allele accuracy.
[4]: the most likely miscalled allele and the proportion of the most likely miscalled allele in all miscalled alleles.

Table S8: The sensitivity (SEN), specificity (SPE), positive predictive value (PPV) and negative predictive value (NPV) calculated from validation samples for each four-digit HLA allele with call threshold 0.5, when STUDY Data of African ancestry were divided to training and validation parts with equal sizes. The SNP markers in the intersect of Illumina platforms were used.

Allele[1]	Num. Train	Freq. Train	Num. Valid.	Freq. Valid.	CR[2] (%)	ACC[3] (%)	SEN (%)	SPE (%)	PPV (%)	NPV (%)	Miscall[4] (%)
HLA–A: Overall accuracy: 100%											
01:01	5	0.0309	4	0.0339	75.0	100.0	100.0	100.0	100.0	100.0	–
02:01	14	0.0864	10	0.0847	80.0	100.0	100.0	100.0	100.0	100.0	–
02:05	4	0.0247	2	0.0169	50.0	100.0	100.0	100.0	100.0	100.0	–
03:01	15	0.0926	9	0.0763	88.9	100.0	100.0	100.0	100.0	100.0	–
23:01	11	0.0679	11	0.0932	90.9	100.0	100.0	100.0	100.0	100.0	–
24:02	3	0.0185	2	0.0169	100.0	100.0	100.0	100.0	100.0	100.0	–
26:01	4	0.0247	3	0.0254	66.7	100.0	100.0	100.0	100.0	100.0	–
29:02	3	0.0185	4	0.0339	25.0	100.0	100.0	100.0	100.0	100.0	–
30:01	14	0.0864	11	0.0932	90.9	100.0	100.0	100.0	100.0	100.0	–
30:02	8	0.0494	11	0.0932	72.7	100.0	100.0	100.0	100.0	100.0	–
33:01	3	0.0185	2	0.0169	100.0	100.0	100.0	100.0	100.0	100.0	–
33:03	13	0.0802	6	0.0508	100.0	100.0	100.0	100.0	100.0	100.0	–
34:02	6	0.0370	6	0.0508	100.0	100.0	100.0	100.0	100.0	100.0	–
36:01	9	0.0556	12	0.1017	91.7	100.0	100.0	100.0	100.0	100.0	–
68:01	5	0.0309	4	0.0339	75.0	100.0	100.0	100.0	100.0	100.0	–
68:02	13	0.0802	10	0.0847	70.0	100.0	100.0	100.0	100.0	100.0	–
HLA–B: Overall accuracy: 96.7%											
07:02	17	0.0850	10	0.0704	30.0	100.0	100.0	100.0	100.0	100.0	–
14:02	2	0.0100	2	0.0141	50.0	100.0	100.0	100.0	100.0	100.0	–
15:03	9	0.0450	8	0.0563	25.0	100.0	100.0	100.0	100.0	100.0	–
18:01	8	0.0400	4	0.0282	25.0	100.0	100.0	100.0	100.0	100.0	–
35:01	14	0.0700	9	0.0634	22.2	100.0	100.0	100.0	100.0	100.0	–
42:01	11	0.0550	6	0.0423	50.0	96.7	100.0	96.3	75.0	100.0	–
45:01	6	0.0300	3	0.0211	100.0	100.0	100.0	100.0	100.0	100.0	–
49:01	6	0.0300	5	0.0352	20.0	100.0	100.0	100.0	100.0	100.0	–
53:01	24	0.1200	21	0.1479	47.6	100.0	100.0	100.0	100.0	100.0	–
57:03	8	0.0400	6	0.0423	16.7	100.0	100.0	100.0	100.0	100.0	–
58:02	8	0.0400	5	0.0352	40.0	100.0	100.0	100.0	100.0	100.0	–
HLA–C: Overall accuracy: 96.5%											
02:02	9	0.0608	9	0.0692	44.4	97.7	50.0	100.0	100.0	98.4	02:10 (100)
02:10	5	0.0338	5	0.0385	20.0	97.7	100.0	97.6	33.3	100.0	–
03:02	4	0.0270	5	0.0385	80.0	100.0	100.0	100.0	100.0	100.0	–
04:01	28	0.1892	25	0.1923	80.0	100.0	100.0	100.0	100.0	100.0	–
06:02	6	0.0405	11	0.0846	63.6	100.0	100.0	100.0	100.0	100.0	–
07:01	24	0.1622	9	0.0692	100.0	100.0	100.0	100.0	100.0	100.0	–
07:02	13	0.0878	8	0.0615	62.5	100.0	100.0	100.0	100.0	100.0	–
08:04	8	0.0541	4	0.0308	50.0	98.8	100.0	98.8	66.7	100.0	–
14:02	3	0.0203	3	0.0231	66.7	100.0	100.0	100.0	100.0	100.0	–
16:01	15	0.1014	20	0.1538	90.0	100.0	100.0	100.0	100.0	100.0	–
17:01	11	0.0743	12	0.0923	91.7	100.0	100.0	100.0	100.0	100.0	–

Continued on next page ...

Table S8 – continued from previous page

Allele[1]	Num. Train	Freq. Train	Num. Valid.	Freq. Valid.	CR[2] (%)	ACC[3] (%)	SEN (%)	SPE (%)	PPV (%)	NPV (%)	Miscall[4] (%)
18:01	5	0.0338	3	0.0231	66.7	100.0	100.0	100.0	100.0	100.0	–
HLA–DRB1: Overall accuracy: 100%											
01:02	5	0.0281	3	0.0208	33.3	100.0	100.0	100.0	100.0	100.0	–
03:01	11	0.0618	9	0.0625	22.2	100.0	100.0	100.0	100.0	100.0	–
03:02	9	0.0506	11	0.0764	27.3	100.0	100.0	100.0	100.0	100.0	–
07:01	19	0.1067	13	0.0903	30.8	100.0	100.0	100.0	100.0	100.0	–
08:04	11	0.0618	11	0.0764	36.4	100.0	100.0	100.0	100.0	100.0	–
09:01	6	0.0337	6	0.0417	33.3	100.0	100.0	100.0	100.0	100.0	–
11:01	13	0.0730	12	0.0833	33.3	100.0	100.0	100.0	100.0	100.0	–
11:02	6	0.0337	5	0.0347	40.0	100.0	100.0	100.0	100.0	100.0	–
13:01	13	0.0730	13	0.0903	15.4	100.0	100.0	100.0	100.0	100.0	–
13:02	10	0.0562	8	0.0556	12.5	100.0	100.0	100.0	100.0	100.0	–
13:03	11	0.0618	8	0.0556	25.0	100.0	100.0	100.0	100.0	100.0	–
15:03	17	0.0955	13	0.0903	30.8	100.0	100.0	100.0	100.0	100.0	–
16:02	3	0.0169	3	0.0208	33.3	100.0	100.0	100.0	100.0	100.0	–
HLA–DQA1: Overall accuracy: 97.2%											
01:01	10	0.0725	9	0.0692	11.1	100.0	100.0	100.0	100.0	100.0	–
01:02	41	0.2971	31	0.2385	35.5	100.0	100.0	100.0	100.0	100.0	–
01:03	10	0.0725	10	0.0769	40.0	100.0	100.0	100.0	100.0	100.0	–
02:01	16	0.1159	16	0.1231	56.2	100.0	100.0	100.0	100.0	100.0	–
04:01	15	0.1087	15	0.1154	33.3	100.0	100.0	100.0	100.0	100.0	–
05:01	14	0.1014	19	0.1462	26.3	97.2	100.0	96.8	83.3	100.0	–
HLA–DQB1 Overall accuracy: 97.7%											
02:01	17	0.1149	16	0.1270	18.8	100.0	100.0	100.0	100.0	100.0	–
03:01	20	0.1351	16	0.1270	25.0	100.0	100.0	100.0	100.0	100.0	–
03:02	7	0.0473	6	0.0476	66.7	100.0	100.0	100.0	100.0	100.0	–
04:02	11	0.0743	10	0.0794	70.0	100.0	100.0	100.0	100.0	100.0	–
05:01	18	0.1216	19	0.1508	47.4	97.7	100.0	97.1	90.0	100.0	–
05:02	6	0.0405	5	0.0397	60.0	100.0	100.0	100.0	100.0	100.0	–
06:02	26	0.1757	18	0.1429	66.7	97.7	91.7	100.0	100.0	99.1	05:01 (100)
06:03	7	0.0473	6	0.0476	16.7	100.0	100.0	100.0	100.0	100.0	–
06:09	4	0.0270	2	0.0159	50.0	100.0	100.0	100.0	100.0	100.0	–
HLA–DPB1: Overall accuracy: 75.0%											
01:01	17	0.1932	16	0.2581	31.2	75.0	100.0	33.3	71.4	100.0	–
04:01	8	0.0909	6	0.0968	16.7	100.0	100.0	100.0	100.0	100.0	–

[1]: the HLA alleles with more than one copy and non-zero sensitivity in the training are listed.
[2]: CR – call rate.
[3]: ACC – allele accuracy.
[4]: the most likely miscalled allele and the proportion of the most likely miscalled allele in all miscalled alleles.

Table S9: The accuracies calculated from the ethnic-specific and multi-ethnic models. For each ethnicity, STUDY Data were divided into training and validation sets with equal sizes. The multi-ethnic model was built using all training samples from multiple ethnicities, whereas the ethnic-specific models were built using the training part of each ethnicity respectively. No call threshold was executed.

	HLA –						
	A	*B*	*C*	*DRB1*	*DQA1*	*DQB1*	*DPB1*
European ancestry							
multi-ethnic model	98.5	96.5	99.1	92.8	97.2	98.7	93.9
ethnic-specific model	98.2	96.6	98.8	92.1	97.3	98.8	93.8
Asian ancestry							
multi-ethnic model	89.1	85.8	95.6	87.2	86.2	96.5	90.4
ethnic-specific model	92.1	87.5	96.6	88.7	86.8	96.0	89.8
Hispanic ancestry							
multi-ethnic model	93.1	77.1	94.4	81.5	96.5	97.7	95.2
ethnic-specific model	93.4	75.0	96.2	82.0	93.8	95.7	93.1
African ancestry							
multi-ethnic model	94.1	81.7	94.6	78.5	79.2	80.2	83.9
ethnic-specific model	92.4	76.8	88.5	77.1	80.0	79.4	74.2
